# Occurrence of *Dickeya* and *Pectobacterium* in Lake Water and the Rhizosphere of Waterside Plants Collected in the French Region La Dombes

**DOI:** 10.3390/microorganisms14071459

**Published:** 2026-07-02

**Authors:** Nicole Hugouvieux-Cotte-Pattat, Véronique Utzinger

**Affiliations:** Microbiology, Adaptation and Pathogeny, Université Lyon 1, INSA-Lyon, CNRS, MAP, UMR 5240, 69622 Villeurbanne, France; veronique.utzinger@univ-lyon1.fr

**Keywords:** *Pectobacteriaceae*, phytopathogenic bacteria, lake water, rhizosphere, bacterial diversity

## Abstract

Pectinolytic bacteria of the *Pectobacteriaceae* family constitute an important group of plant pathogens. Apart from infected plants, they are regularly found in aquatic environments. Pectinolytic isolates were selected, during 3 years, from water of naturally eutrophic lakes and from the rhizosphere of waterside plants in a site protected from direct agricultural inputs. A total of 89 isolates were assigned to *Enterobacterales* including the genera *Dickeya* (62%) and *Pectobacterium* (13%). In contrast to previous reports showing the prevalence of *Pectobacterium* species in river water, the *Dickeya* isolates were largely preponderant in lake water. Six *Dickeya* and four *Pectobacterium* species were isolated from water including *D. oryzae*, *D. lacustris*, *D. parazeae*, *P. quasiaquaticum* and *P. aquaticum* and, at a low frequency, *D. chrysanthemi*, *D. aquatica*, *D. zeae*, *P. brasiliense*, and *P. versatile*. The most common species isolated from plant rhizosphere was *D. chrysanthemi*. Notably, the rhizosphere of *Solanum dulcamara* harbored the highest number and diversity of *Dickeya* and *Pectobacterium* isolates. Members of different species and/or genera were found in the same water sample or plant rhizosphere, indicating that they can cohabit in close environments. Despite noticeable individual variations, the water strains have a pathogenic potential similar to that of other strains of the same species.

## 1. Introduction

Pectinolytic bacteria of the *Pectobacteriaceae* family, in the *Enterobacterales* order, constitute an important group of plant pathogens able to infect a wide range of hosts, monocotyledons or dicotyledons, including herbaceous and woody plants [1,2]. The genera *Dickeya*, *Musicola* and *Pectobacterium* are often collectively designed soft-rot *Pectobacteriaceae* (SRP). SRPs were listed in the “top 10” plant pathogenic bacteria, based on their economic and scientific importance [3]. To degrade pectin, SRPs secrete a large array of enzymes, including very efficient pectate-lyases [4]. Pectin, one of the major plant cell wall polysaccharides, is necessary for the cohesion of the plant tissue. Pectin degradation leads to plant tissue maceration, and the usual symptom on infected plants is a soft rotting [1]. In 2005, a major classification change was undertaken in the non-homogeneous genus *Erwinia*, with the assignment of *E. chrysanthemi* and *Brenneria paradisiaca* to the novel genus *Dickeya* [5]. Members of the species *D. paradisiaca* were recently reassigned to the genus *Musicola* [6]. After careful revision of heterogeneous species and description of new isolates, the genus *Dickeya* currently comprises thirteen species with validly published names: *D. ananatis*, *D. aquatica*, *D. chrysanthemi*, *D. dadantii*, *D. dianthicola*, *D. fangzhongdai*, *D. lacustris*, *D. oryzae*, *D. parazeae*, *D. poaceaphila*, *D. solani*, *D. undicola*, and *D. zeae* [5,7,8,9,10,11,12,13,14,15,16]. The genus *Pectobacterium* is also highly heterogeneous, and its classification has been subject to extensive revision over the last decade, giving rise to 22 species with validly published names [11,17,18,19] (https://lpsn.dsmz.de/genus/pectobacterium, accessed on 1 April 2026).

Because many SRP hosts are economically important crops, SRPs have been investigated primarily from an agronomic perspective. Consequently, most SRP strains available in laboratory collections have been isolated from diseased plants [20]. Some earlier reports relate their presence in non-host environments, mainly in water [21], but the taxonomic status of strains isolated during the 1980s remains uncertain due to advances in SRP classification [15,22]. In addition, some water-associated SRP species were recently described, such as *D. aquatica* [7], *D. lacustris* [10], *D. undicola* [11], *P. aquaticum* [17], *P. quasiaquaticum* [19], *P. polonicum* [23], and *P. fontis* [24]. Since the relative concentration of SRPs among numerous environmental bacteria is very low, metagenomic approaches were shown to lack sensitivity to identify SRPs in surface water [25]. Thus, the exploration of SRPs in aquatic ecosystems requires a specific selection based of their high pectinolytic activity. This could be achieved using the semi-selective CVP medium developed to isolate SRPs from diseased plants [26]. This method was used in recent studies to identify SRPs present in aquatic environments [25,27,28]. Recently, a large survey of SRPs in the river Durance in the South of France led to the identification of several *Pectobacterium* or *Dickeya* isolates [28]. In this river, the abundance of SRPs correlated with the agricultural gradient, suggesting contaminations from adjacent cultured fields. The majority of isolates were assigned to the genus *Pectobacterium*, mostly to the species *P. aquaticum* and *P. versatile*, and less than 6% of the isolates belonged to the genus *Dickeya*, mostly to the species *D. oryzae* [28].

Although the SRP presence in surface waters has been known for a long time, data on plant isolates remained far more abundant than those concerning environmental isolates. Our objective was to explore the SRP diversity in other types of aquatic ecosystems, including stagnant lake water and the rhizosphere of riparian plants. To avoid contamination from infected crops, we performed SRP surveys in a zone protected from direct agricultural inputs, a conservatory site reserved for the study and the preservation of biodiversity, including naturally eutrophic lakes, in a wetland region called La Dombes in France. Bacteria present in lake water or in the rhizosphere of waterside plants were selected on the basis of their pectinolytic activity, using either direct spreading on the semi-selective medium CVP [26] or spreading on this medium after growth in a SRP enrichment broth [29]. Our three-year study provides information on SRP community composition, abundance and diversity. It shows that SRPs, and especially *Dickeya* species, are common in this type of aquatic environment, independently of agricultural contaminations.

## 2. Materials and Methods

### 2.1. Bacterial Strains, Media, and Culture Conditions

Bacterial strains used for detailed phenotypic analyses are listed in Table S1. All SRP strains were grown at 30 °C in rich medium LB or in minimal medium M63 [30] containing a carbon source at the final concentration of 2 g·L^−1^ (glycerol, D-glucose, D-xylose, D-arabinose, mannitol, melibiose, raffinose or trehalose). Except when indicated, 15 g·L^−1^ agar was added for solid media.

Pectinolytic bacteria were selected on plates containing the crystal violet pectate (CVP) medium [26]. We used single layer CVP containing 18 g·L^−1^ sodium polygalacturonate (dipecta), 4 g·L^−1^ agar, 5 g·L^−1^ tri-sodium citrate, 2 g·L^−1^ NaNO3, 1 g·L^−1^ tryptone, 1 g·L^−1^ CaCl_2_·2H_2_O and 15 mg·L^−1^ crystal violet and adjusted to a pH of 7. CVP is a semi-selective medium containing pectin and citrate as carbon sources and crystal violet which inhibits the growth of Gram-positive bacteria. Due to their high capacity to degrade pectin, SRP colonies form characteristic cavities when grown on CVP medium. For enrichment in SRPs, we used a pectate enrichment broth (PEB) containing 2.6 g·L^−1^ sodium polygalacturonate, 1 g·L^−1^ (NH_4_)_2_SO_4_, 1 g·L^−1^ K_2_HPO_4_, and 0.3 g·L^−1^ MgSO_4_.7H_2_O and adjusted to a pH of 7 [29]. This medium favors the growth of bacteria able to use polygalacturonate as the sole carbon source.

### 2.2. Collection of Water Samples and Riparian Plant Rhizosphere

Samples were collected in a reserve (244 ha) located in La Dombes (latitude 45.951937, longitude 4.883082, altitude 290 m), belonging to the Pierre Verot Foundation (https://www.fondation-pierre-verots.fr/). It includes four shallow lakes, Boufflers (28 ha), Praillebard (22 ha), Riquet (5 ha), and Page (3.2 ha) with an average depth of 1–2 m, and maximum of 10 m. Water samples (50 mL) were taken approximately 1 m from the edge, in surface water (about 0.5 m deep). Sampling was performed in the morning, and the samples were treated in the laboratory in the early afternoon. The rhizosphere of waterside plants was collected by extracting plants whose roots soaked at least partially in water. Plant roots (about 10 cm) were immediately placed in 50 mL distilled water and kept for 24 h at room temperature before treatment.

Water samples from lakes or from 24 h soaked roots were similarly submitted to the following treatments. First, each sample was filtered on a pleated paper filter to remove suspended particles. This filtration allowed the elimination of algae, protists and filamentous fungi. To estimate population of cultivable bacteria, appropriate dilutions of filtered water were spread on LB plates. After incubation 48 h at 30 °C, colony counts were used to determine the number of colony forming units per mL (CFU/mL).

### 2.3. Sample Processing for Isolation of Pectinolytic Bacteria

To detect pectinolytic bacteria, 200 μL of each filtered water sample was spread on CVP medium. Spreading on CVP was also performed after sample concentration (up to 30-fold). Usually, bacteria present in the 15 mL sample were recovered in the pellet after centrifugation for 10 min at 7000 g. A total of 10 μL and 100 μL of the concentrated sample was spread on CVP medium. In parallel, 100 μL of concentrated sample was inoculated into 2 mL of enrichment medium, PEB. After incubation for 48 h at 30 °C, the PEB cultures were serially diluted in water before spreading appropriate dilutions on CVP medium.

The CVP plates were incubated at 30 °C and examined for the presence of pit-forming colonies each day during one week. For each sample, several pit-forming colonies were picked randomly, favoring those with different appearances on CVP plates, in terms of cavity size and depth, as well as colony aspect, mucoidy and color. To limit the recovery of clonal bacteria, a reduced number of colonies was taken from each sample. The selected pectinolytic colonies were picked up in 50 μL water, appropriately diluted in water before spreading appropriate dilutions on CVP and LB media to obtain isolated bacterial colonies. A repeated isolation step on LB plates allowed the obtention of pure isolates.

### 2.4. Phenotypic Analysis of Pectinolytic Isolates

The phenotype of the purified pectinolytic colonies was analyzed by replica plating on different media to evaluate the secretion of plant cell wall-degrading enzymes (pectinases, cellulases, proteases) and the utilization of a set of carbon sources (glycerol, sucrose, mannitol, melibiose, raffinose, D-arabinose, xylose, trehalose) [10]. For the detection of pectinase activity, bacteria were grown on M63 agar plates supplemented with 2 g·L^−1^ glycerol and 4 g·L^−1^ polygalacturonic acid. After incubation for 24 h at 30 °C, plates were flooded with a saturated solution of copper acetate. Clear haloes appear around colonies secreting pectate-lyases. Cellulase activity was detected on M63 agar plates supplemented with 2 g·L^−1^ glycerol and 10 g·L^−1^ carboxymethylcellulose. After incubation for 24 h at 30 °C, the plates were overflowed with 10 mg mL^−1^ Congo red solution for 10 min. After 5 min washing with 1 M NaCl, clear haloes surrounded colonies secreting cellulases. Protease secretion was tested on LB plates supplemented with skim milk (12.5 g·L^−1^). After incubation at 30 °C for 24 to 48 h, clear haloes were visible around colonies secreting proteases.

The motility of the isolates was tested on semisolid agar plates. To assay the swimming motility inside the medium, the bacterial strains were inoculated by picking colonies into 3 g·L^−1^ agar LB plates. The swarming motility, at the surface of medium, was assayed by depositing 2 μL of bacterial cultures grown in LB onto 6 g·L^−1^ agar LB plates supplemented with 5 g·L^−1^ glucose. Plates were incubated at 30 °C. The diameter of the growth zone was measured at 16, 24 and 48 h and taken as a measure of the bacterial motility. The experiment was performed twice, with 3 replicates per experiment.

### 2.5. PCR Amplification and Sequencing

In addition to phenotypic classification, our identification relied primarily on the use of the partial sequence (804 nt) of the housekeeping gene *gapA*. This method proved highly relevant for studying the diversity of SRPs isolated from various host and non-host plants, as well as from soil and surface water samples [28,31].

The purified isolates were subjected to PCR on colonies using primers specific for the gene *gapA* (gapAF AAGTGAAAGACGGTCACCTGGT and gapAR CGATCAGGTCCAGAACCTTGTT, 0.8 kb amplicon) [31]. The PCR mixture contained 1.5 mM MgCl_2_ in 10 mM Tris-HCl pH 9, 0.2 mM of each dNTP, 0.4 mM of each primer, and 2.5 U Taq polymerase, in a final volume of 25 μL (Illustra PuRe Taq^TM^ Ready to Go^TM^, GE Healthcare, Buc, France). PCR cycling parameters were initial denaturation at 95 °C for 5 min, followed by 35 cycles of 95 °C for 1 min, 55 °C for 1 min and 72 °C for 1 min, with a final extension of 5 min at 72 °C. Amplicons were sequenced in both directions, with the two *gapA* primers, using a commercial sequencing service (Microsynth France, Vaulx-en-Velin, France). To further clarify the SRP identity and phylogeny, phylogenetic trees were generated using a ready-to-use pipeline operating with programs MUSCLE 3.7 for multiple alignment, PhyML 3.0 for tree building, and TreeDyn 198.3 for tree rendering [32] (Phylogeny.lirmm.fr, accessed on 1 April 2026).

We also used genomic analysis to verify the taxonomic assignment of some isolates, two from water samples and six from the rhizosphere of *S. dulcamara*. These strains belong to the species *D. aquatica*, *D. chrysanthemi*, *D. lacustris*, *D. oryzae*, *D. parazeae*, *P. brasiliense* and *P. versatile* (Figure S1). In each case, the calculation of ANI and dDDH values with the type strains of the different *Dickeya* or *Pectobacterium* species confirmed the initial identification using *gapA*.

### 2.6. Maceration of Plant Tissues

The maceration capacity of the strains was tested using whole potato tubers and chicory leaves, as previously described [10]. In brief, the potato tubers were washed in tap water and dried. A small hole was made with a sterile pipette tip on potato tubers, and inoculated with 5 μL of the bacterial suspension (5 × 10^6^ CFU). Inoculated holes were then covered with mineral oil to provide anaerobic conditions. After 48 h at 30 °C, the rotted potato tissue was recovered and weighed to estimate the disease severity. Infection of chicory leaves was performed by depositing 10 μL of a bacterial suspension (10^6^ CFU) on a small wound made with a sterile pipette tip in the center of each leaf. After incubation in a dew chamber for 24 h at 30 °C, the length of rotted tissue was measured to estimate the disease severity. At least 6 potato tubers and 10 chicory leaves were used to evaluate the maceration capacity of each tested strains. These experiments were performed twice. Data are presented as means ± standard error.

## 3. Results

### 3.1. Collection of Water and Rhizosphere Samples

The samples were collected in a nature reserve representative of a temperate freshwater wetland. This reserve is located at the top of a watershed, away from any effluent coming from agricultural lands. Water samples were collected from four lakes surrounded by wetlands and fed only by rainwater. Surface water was collected from the lake shore during six surveys performed during three successive years (2017 to 2019), in June, July, August, September, October or March (Table 1). As expected, the sampling season affected water temperature, which ranged from 15.6 to 29.2 °C in summer (June to September), 13.7 to 14 °C in autumn (October) and 3.4 to 4.8 °C in winter (March) (Table 1). A total of 70 water samples were collected. The concentrations of cultivable bacteria observed on LB plates were highly variable, with extreme values ranging from 2 × 10^2^ CFU/mL in March to 5 × 10^4^ CFU/mL in August (Table 1). Since LB medium tends to favor fast-growing copiotrophic bacteria, such as *Pectobacteriaceae*, these CFU values may underestimate the total cultivable bacterial community.

During the first survey performed in June 2017, some water samples were collected from small ponds, ditches or pools. As no pectinolytic bacteria were detected in these samples, the corresponding water systems were no more studied. However, roots of various riparian plants were collected during the next surveys (Table 1). The plant roots were soaked in sterile water to assess the presence of pectinolytic bacteria in their rhizosphere. A total of 46 rhizosphere samples were collected from different plants, including 22 bittersweets (*Solanum dulcamara*), 10 common rushes (*Juncus effusus*), 10 reeds (*Phragmites australis*) and 4 Bidens (*Bidens tripartite*, a floating plant) (Table 1).

**Table 1:** Information on the sampling surveys.

Sampling Day	27 June	4 July	27 August	12 September	9 October	20 March	
	2017	2018	2019	2017	2018	2018	
	Summer	Summer	Summer	Summer	Automn	Winter	Total
Air temperatures	27–29 °C	25–27 °C	22–27 °C	17–19 °C	14–15 °C	1–2 °C	
Number of water samples	8	14	12	15	9	12	70
Water temperatures	27.2–29.2 °C	24.7–28.8 °C	20.5–25 °C	15.6–18.9 °C	13.7–14 °C	3.4–4.8 °C	
Cultivable bacteria on LB plates (CFU/mL)	8 × 10^2^–4 × 10^4^	1.3 × 10^3^–2 × 10^4^	10^3^–5 × 10^4^	3 × 10^2^–2 × 10^4^	1.3 × 10^3^–4 × 10^4^	2 × 10^2^–1.3 × 10^4^	
Pectinolytic colonies on CVP per liter	0–4 × 10^3^	0–6 × 10^3^	0–10^4^	0–2 × 10^3^	0–10^3^	0–10^3^	
Number of samples containing pectinolytic bacteria	8	8	8	9	7	4	44
Number of pectinolytic colonies isolated	40	50	70	30	15	40	245
Number of rhizosphere samples	0	5	16	3	13	9	46
Collected plants		Bittersweet	Bittersweet, reed, rush, bidens	Bittersweet	Bittersweet, reed, rush	Bittersweet, reed, rush, bidens	
Number of samples containing pectinolytic bacteria		4	5	3	6	7	25
Plant rhizospheres including SRP		Bittersweet	Rush, bidens	Bittersweet		Rush	Bittersweet, rush, bidens
Number of pectinolytic colonies isolated		9	29	9	6	35	88

### 3.2. Detection of Pectinolytic Bacteria on Semi-Selective Medium

Due to their ability to degrade the gelling agent pectin, pectinolytic colonies form characteristic pits on CVP plates. CVP was developed to isolate SRPs from diseased plants that contain a large density of the pathogen. As it contains citrate, CVP allows growth of several environmental bacteria. Using the water samples, we observed a high number of colonies growing on CVP plates. The number of colonies observed on CVP was usually higher than 10^2^ CFU/mL and reached up to 8 × 10^2^ CFU/mL in summer samples. The high density of colonies covering the CVP surface sometimes hampered the isolation of pectinolytic clones. We limited the sample concentration to 30-fold, to increase the proportion of pit-forming colonies that could be successfully isolated. Under these experimental conditions, the detection threshold of pectinolytic bacteria on CVP was about 66 bacteria per liter of water (1 bacteria per 15 mL). Population density was below the detection threshold for 26 of the 70 water samples. However, pit-forming colonies were observed in 63% of the samples (44 out of 70). In these samples, the concentration of pit-forming colonies was estimated to range from 66 to 10^4^ colonies per liter of water (Table 1). For the rhizosphere samples, pit-forming colonies were observed in 14 out of 46 samples (30%), including rhizospheres from 8 bittersweets (out of 22), 3 rushes (out of 10), 1 Bidens (out of 4) and 2 reeds (out of 10). The medium used for the enrichment step contained only polygalacturonate as a carbon source, but many bacteria were recovered after inoculation of water or rhizosphere samples into PEB, maybe due to the utilization of pectic oligomers released by pectinolytic bacteria. In each case, the high number of colonies growing on CVP was a technical limitation to recover pectinolytic bacteria from these complex environmental samples.

In parallel, we checked whether each of the 13 characterized *Dickeya* species could be easily detected using CVP, by plating dilutions of type strains of each species on this medium. Colonies from eleven species, namely *D. ananatis*, *D. aquatica*, *D. chrysanthemi*, *D. dadantii*, *D. fangzhongdai*, *D. lacustris*, *D. oryzae*, *D. parazeae*, *D. solani*, *D. undicola*, and *D. zeae*, formed deep pits on CVP. In contrast, colonies of *D. dianthicola* and *D. poaceaphila* gave barely visible cavities, indicating that these two species may be difficult to recover from environmental samples. Concerning the *Pectobacterium* species, we observed deep pits on CVP with *P. atrosepticum*, *P. aquaticum*, *P. betavascularum*, *P. brasiliense*, *P. carotovorum*, *P. odoriferum*, *P. peruviense*, *P. polaris*, *P. quasiaquaticum* and *P. versatile*. Other species were not tested. We also verified that these SRP species grow in the enrichment medium PEB which contains polygalacturonate as the sole carbon source, except *D. poaceaphila* which was already shown to be unable to use polygalacturonate as a carbon source for growth [12].

### 3.3. Classification of the Pectinolytic Isolates by Phenotypic Analysis

Pectinolytic isolates were picked from CVP and purified by plating successive dilutions on CVP and LB medium. In a first step, a total of 245 and 88 pectinolytic clones were isolated from water samples and rhizosphere samples, respectively (Table 1). Phenotypic tests were performed to obtain a first view on their diversity. As SRPs are characterized by the secretion of plant cell wall-degrading enzymes, we used in situ tests for the detection of pectinase, cellulase, and protease activities (Table 2). The plate assay used for pectinase detection is largely less sensitive than CVP plates used for the isolation of pectinolytic colonies. It is commonly used for SRP analysis, but a large proportion of pectinolytic isolates from CVP gave a negative result with the pectinase plate assay. In accordance with the colony aspect (color, size, etc.) and growth characteristics (see below), such isolates were considered as non-SRP and discarded. An historical classification of *Dickeya* members in biovars was based on their capacity to growth with a set of carbon sources, mostly sugars [5]. Analysis of pectinolytic isolates revealed different profiles using the following sugars: D-arabinose, melibiose, mannitol, xylose and trehalose (Table 2). A total of 42 pectinolytic isolates belonged to the *Dickeya* biovar 3/8 characterized by the ability to assimilate D-arabinose (Table 2); this biovar could correspond to six species, namely *D. dadantii*, *D. fangzhongdai*, *D. oryzae*, *D. parazeae*, *D. solani*, and *D. zeae*. A total of 16 pectinolytic isolates were members of biovar 10, recently proposed for the two species *D. aquatica* and *D. lacustris* [10] and differing by the inability to assimilate xylose (Table 2). Eight pectinolytic isolates answered like biovar 5/6, described for *D. chrysanthemi* [5]. The other biochemical profiles did not correspond to the previously described *Dickeya* biovars, suggesting that they belong to other pectinolytic genera, such as *Pectobacterium*.

### 3.4. Taxonomic Affiliation of Pectinolytic Isolates from Water Samples

**Figure 1:**
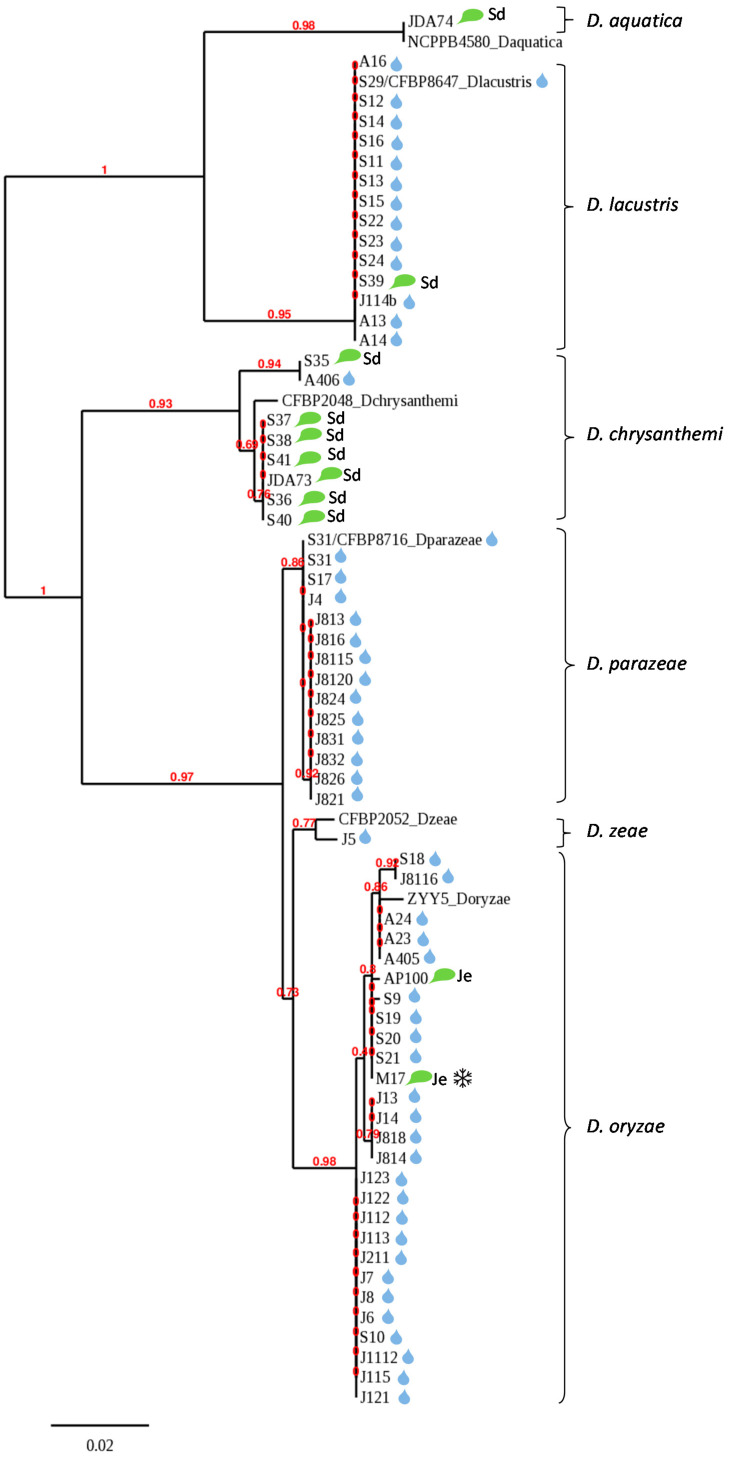
The *gapA* phylogenetic tree of *Dickeya* isolates. The tree was generated using a ready-to-use pipeline operating with programs MUSCLE for multiple alignment, PhyML for tree building, and TreeDyn for tree rendering (Phylogeny.lirmm.fr). Bootstrap values are shown above the corresponding branch. The bar indicates the number of changes per nucleotide position. The species name is given for each type strain. For clarity, only the type strains corresponding to the species isolated in this study are included in the tree. The drop or the leaf after the isolate designation indicate a water or rhizosphere origin, respectively. For rhizosphere isolates, the corresponding plant is indicated near the leaf: Sd, *Solanum dulcamara*, and Je, *Juncus effusus.* The snowflake indicates the strain isolated in winter.

**Figure 2:**
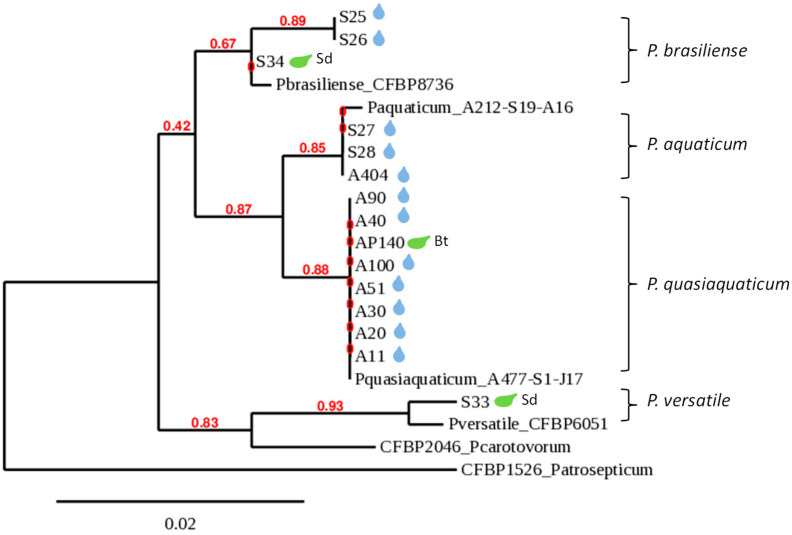
The *gapA* phylogenetic tree of *Pectobacterium* isolates. Legend as in Figure 1.

In order to identify the pectinolytic isolates, we used the partial sequence of the housekeeping gene *gapA*, shown to be a reliable phylogenetic marker for SRPs [31]. After PCR amplification, high-quality sequences were obtained for 89 isolates from water and 24 pectinolytic isolates from rhizosphere samples. The *gapA* phylogenetic analysis suggested that the 89 isolates from water samples consist in 55 *Dickeya* strains (62%), 12 *Pectobacterium* strains (13%), and 22 isolates from other *Enterobacterales* families (25%) (Table 3). In the *gapA* phylogenetic trees, the SRP isolates were distributed into different clades, each including a type strain (Figure 1 and Figure 2). The 55 *Dickeya* isolates were thus assigned to five characterized species, namely *D. oryzae* (26), *D. lacustris* (14), *D. parazeae* (13), *D. chrysanthemi* (1), and *D. zeae* (1) (Figure 1). The 12 *Pectobacterium* isolates were distributed in three species, *P. quasiaquaticum* (7), *P. aquaticum* (3), and *P. brasiliense* (2) (Figure 2). For the 22 other *Enterobacterales* isolates, the *gapA* sequences suggest that they belong to the genera *Kosakonia*, *Klebsiella*, *Serratia*, *Lelliottia*, and *Enterobacter* (Table 3 and Table S2). In comparison to the deep cavities formed on CVP by *Dickeya* or *Pectobacterium* members, the non-SRP *Enterobacterales* strains usually generate large but shallow cavities (Table 3).

When individual water samples were considered, we observed that several samples contained two SRP genera. In the September survey, a sample from lake Boufflers contained both *D. lacustris* and *P. brasiliense*, and another sample from this lake contained *D. lacustris*, *D. oryzae* and *P. aquaticum.* In the August survey of lake Praillebard, a sample contained *D. lacustris* and *P. quasiaquaticum*, another sample contained *D. oryzae* and *P. quasiaquaticum*, and a third sample contained *D. chryanthemi*, *D. oryzae*, *P. aquaticum*, and *P. quasiaquaticum.* Other samples enclosed two *Dickeya* species, such as *D. lacustris* with *D. oryzae*, *D. oryzae* with *D. parazeae*, or *D. lacustris* with *D. parazeae* (Table 3 and Table 4).

**Table 3:** The 89 water isolates identified by phenotypic analysis and *gapA* sequencing.

Isolate Name	Laboratory Collection	Date	Lake	Water Temp. °C	Selection Method	Aspect of Pits on CVP	Biovar	Identification by *gapA* Sequence
J4		June 2017	Boufflers	27.2	filtered water	large, deep	Bv 3/8	*Dickeya parazeae*
J5	A6252	June 2017	Boufflers	27.2	filtered water	deep	Bv 3/8	*Dickeya zeae*
J6	A6253	June 2017	Riquet	28.6	concentration	deep	Bv 3/8	*Dickeya oryzae*
J7		June 2017	Riquet	28.6	concentration	deep	Bv 3/8	*Dickeya oryzae*
J8		June 2017	Riquet	28.6	concentration	deep	Bv 3/8	*Dickeya oryzae*
J9		June 2017	Riquet	29.2	concentration	deep	_	*Serratia oryzae*
J10		June 2017	Riquet	29.2	enrichment	shallow	_	*Klebsiella quasipneumoniae*
J11		June 2017	Riquet	29.2	enrichment	shallow	_	*Klebsiella quasipneumoniae*
J12		June 2017	Riquet	29.2	enrichment	shallow	_	*Klebsiella quasipneumoniae*
J13		June 2017	Riquet	29.2	filtered water	deep	Bv 3/8	*Dickeya oryzae*
J14		June 2017	Riquet	29.2	filtered water	deep	Bv 3/8	*Dickeya oryzae*
S9		September 2017	Page	18	concentration	large, deep	Bv 3/8	*Dickeya oryzae*
S10		September 2017	Page	18	concentration	large, deep	Bv 3/8	*Dickeya oryzae*
S11		September 2017	Page	18	enrichment	narrow, deep	Bv 10	*Dickeya lacustris*
S12	A6599	September 2017	Page	18	enrichment	narrow, deep	Bv 10	*Dickeya lacustris*
S13		September 2017	Page	18	enrichment	narrow, deep	Bv 10	*Dickeya lacustris*
S14		September 2017	Page	18	enrichment	narrow, deep	Bv 10	*Dickeya lacustris*
S15	A6254	September 2017	Page	18	enrichment	narrow, deep	Bv 10	*Dickeya lacustris*
S16	A6600	September 2017	Praillebard	15.6	concentration	narrow, deep	Bv 10	*Dickeya lacustris*
S17	A6601	September 2017	Praillebard	15.6	enrichment	large, deep	Bv 3/8	*Dickeya parazeae*
S18	A6256	September 2017	Boufflers	18.2	enrichment	large, deep	Bv 3/8	*Dickeya oryzae*
S19		September 2017	Boufflers	18.2	enrichment	large, deep	Bv 3/8	*Dickeya oryzae*
S20	A6066	September 2017	Boufflers	17.7	enrichment	large, deep	Bv 3/8	*Dickeya oryzae*
S21		September 2017	Boufflers	17.7	enrichment	large, deep	Bv 3/8	*Dickeya oryzae*
S22		September 2017	Boufflers	17.4	enrichment	narrow, deep	Bv 10	*Dickeya lacustris*
S23		September 2017	Boufflers	17.5	enrichment	narrow, deep	Bv 10	*Dickeya lacustris*
S24	A6255	September 2017	Boufflers	17.5	enrichment	narrow, deep	Bv 10	*Dickeya lacustris*
S25	A6282	September 2017	Boufflers	17.5	enrichment	deep	_	*Pectobacterium brasiliense*
S26		September 2017	Boufflers	17.5	enrichment	deep	_	*Pectobacterium brasiliense*
S27		September 2017	Boufflers	18.9	concentration	deep	Bv 7/9	*Pectobacterium aquaticum*
S28	A6073	September 2017	Boufflers	18.9	concentration	large, deep	Bv 7/9	*Pectobacterium aquaticum*
S29	A6067	September 2017	Boufflers	18.9	enrichment	narrow, deep	Bv 10	*Dickeya lacustris*
S30		September 2017	Boufflers	18.9	enrichment	large, deep	Bv 3/8	*Dickeya oryzae*
S31	A6068	September 2017	boufflers	17.8	enrichment	deep	Bv 3/8	*Dickeya parazeae*
S32	A6074	September 2017	Boufflers	18.6	filtered water	shallow	_	*Serratia oryzae*
M6		March 2018	Riquet	4.8	concentration	large, shallow		*Serratia* sp.
M10		March 2018	Riquet	3.4	concentration	deep		*Serratia* sp.
J112		July 2018	Page	24.7	concentration	deep	Bv 3/8	*Dickeya oryzae*
J113		July 2018	Page	24.7	concentration	deep	Bv 3/8	*Dickeya oryzae*
J114	A6194	July 2018	Page	24.7	concentration	deep	Bv 10	*Dickeya lacustris*
J115		July 2018	Page	24.7	concentration	deep	Bv 3/8	*Dickeya oryzae*
J1112		July 2018	Page	24.7	concentration	deep	Bv 3/8	*Dickeya oryzae*
J121		July 2018	Page	25.3	concentration	deep	Bv 3/8	*Dickeya oryzae*
J122		July 2018	Page	25.3	concentration	deep	Bv 3/8	*Dickeya oryzae*
J123		July 2018	Page	25.3	concentration	deep	Bv 3/8	*Dickeya oryzae*
J132		July 2018	Page	23.9	concentration	shallow	_	*Kosakonia cowanii*
J211		July 2018	Page	25	concentration	deep	Bv 3/8	*Dickeya oryzae*
J711		July 2018	Boufflers	26.4	concentration	large, shallow	_	*Kosakonia cowanii*
J712		July 2018	Boufflers	26.4	enrichment	large, shallow	_	*Klebsiella pasteurii*
J811		July 2018	Riquet	28.3	concentration	shallow	_	*Kosakonia cowanii*
J813	A6257	July 2018	Riquet	28.3	concentration	deep	Bv 3/8	*Dickeya parazeae*
J814	A6258	July 2018	Riquet	28.3	concentration	deep	Bv 3/8	*Dickeya oryzae*
J816		July 2018	Riquet	28.3	concentration	deep	Bv 3/8	*Dickeya parazeae*
J818		July 2018	Riquet	28.3	concentration	deep	Bv 3/8	*Dickeya oryzae*
J821		July 2018	Riquet	27.5	filtered water	deep	Bv 3/8	*Dickeya parazeae*
J824		July 2018	Riquet	27.5	filtered water	deep	Bv 3/8	*Dickeya parazeae*
J825		July 2018	Riquet	27.5	concentration	shallow	Bv 3/8	*Dickeya parazeae*
J826		July 2018	Riquet	27.5	concentration	deep	Bv 3/8	*Dickeya parazeae*
J8115		July 2018	Riquet	28.2	enrichment	deep	Bv 3/8	*Dickeya parazeae*
J8116		July 2018	Riquet	28.2	enrichment	deep	Bv 3/8	*Dickeya oryzae*
J8120		July 2018	Riquet	28.2	enrichment	deep	Bv 3/8	*Dickeya parazeae*
J831		July 2018	Riquet	28.8	enrichment	deep	Bv 3/8	*Dickeya parazeae*
J832		July 2018	Riquet	28.8	enrichment	deep	Bv 3/8	*Dickeya parazeae*
P62C		October 2018	Boufflers	14	concentration	shallow	_	*Lelliottia* sp.
P64		October 2018	Boufflers	14	filtered water	shallow	_	*Klebsiella pasteurii*
PE64		October 2018	Boufflers	14	enrichment	shallow	_	*Klebsiella pasteurii*
P8R		October 2018	Riquet	13.7	filtered water	shallow	_	*Serratia marcescens*
A10		August 2019	Praillebard	20.5	concentration	deep	_	*Enterobacter* sp.
A11		August 2019	Praillebard	20.5	concentration	deep	Bv 7/9	*Pectobacterium quasiaquaticum*
A12		August 2019	Praillebard	20.5	concentration	shallow	_	*Klebsiella grimontii*
A13		August 2019	Praillebard	20.5	enrichment	deep	Bv 10	*Dickeya lacustris*
A14	A6337	August 2019	Praillebard	20.5	enrichment	deep	Bv 10	*Dickeya lacustris*
A15		August 2019	Praillebard	20.5	enrichment	shallow	_	*Kosakonia sacchari*
A16	A6338	August 2019	Praillebard	20.5	enrichment	narrow, deep	Bv 10	*Dickeya lacustris*
A20		August 2019	Praillebard	21.2	concentration	large, deep	Bv 7/9	*Pectobacterium quasiaquaticum*
A23	A6339	August 2019	Praillebard	21.2	concentration	narrow, deep	Bv 3/8	*Dickeya oryzae*
A24	A6340	August 2019	Praillebard	21.2	filtered water	narrow, deep	Bv 3/8	*Dickeya oryzae*
A25		August 2019	Praillebard	21.2	enrichment	shallow	_	*Kosakonia sacchari*
A30		August 2019	Praillebard	21.8	concentration	large, deep	Bv 7/9	*Pectobacterium quasiaquaticum*
A40	A6344	August 2019	Praillebard	22.2	filtered water	large, deep	Bv 7/9	*Pectobacterium quasiaquaticum*
A404	A6345	August 2019	Praillebard	22.2	concentration	narrow, deep	Bv 7/9	*Pectobacterium aquaticum*
A405	A6341	August 2019	Praillebard	22.2	concentration	narrow, deep	Bv 7/9	*Dickeya oryzae*
A406	A6342	August 2019	Praillebard	22.2	concentration	narrow, deep	Bv 7/9	*Dickeya chrysanthemi*
A407		August 2019	Praillebard	22.2	enrichment	shallow	_	*Kosakonia sacchari*
A408		August 2019	Praillebard	22.2	enrichment	shallow	_	*Kosakonia sacchari*
A51		August 2019	Praillebard	22.9	filtered water	deep	Bv 7/9	*Pectobacterium quasiaquaticum*
A63		August 2019	Praillebard	22.9	enrichment	deep	_	*Kosakonia sacchari*
A90		August 2019	Praillebard	25	concentration	large, deep	Bv 7/9	*Pectobacterium quasiaquaticum*
A100		August 2019	Praillebard	24.9	concentration	large, deep	Bv 7/9	*Pectobacterium quasiaquaticum*

**Table 4:** Samples containing more than one SRP species and/or SRP genera.

Sample Information			Identified SRP	
Date	Lake	Water Temperature or Plant	*Dickeya* Species	*Pectobacterium* Species
September 2017	Page	18 °C	*D. lacustris*, *D.oryzae*	
July 2018	Page	24.7 °C	*D. lacustris*, *D. oryzae*	
July 2018	Riquet	28.3 °C	*D. oryzae*, *D. parazeae*	
July 2018	Riquet	28.2 °C	*D. oryzae*, *D. parazeae*	
June 2017	Boufflers	27.2 °C	*D. parazeae*, *D. zeae*	
September 2017	Boufflers	17.5 °C	*D. lacustris*	*P. brasiliense*
September 2017	Boufflers	18.9 °C	*D. lacustris*, *D. oryzae*	*P. aquaticum*
September 2017	Praillebard	15.6 °C	*D. lacustris*, *D. parazeae*	
August 2019	Praillebard	20.5 °C	*D. lacustris*	*P. quasiaquaticum*
August 2019	Praillebard	21.2 °C	*D. oryzae*	*P. quasiaquaticum*
August 2019	Praillebard	22.2 °C	*D. chrysanthemi*, *D. oryzae*	*P. aquaticum*, *P. quasiaquaticum*
September 2017	Riquet	Bittersweet DA3	*D. chrysanthemi*, *D. lacustris*	
September 2017	Boufflers	Bittersweet DA2	*D. chrysanthemi*	*P. brasiliense*
July 2018	Boufflers	Bittersweet DA7	*D. aquatica*, *D. chrysanthemi*	

### 3.5. Effect of the Selection Method on SRP Recovery from Water Samples

To detect SRPs on CVP, we directly spread the filtered water sample, not concentrated and after concentration. We also used an enrichment step, consisting of plating on CVP after growth in PEB. For water samples, most SRP isolates were recovered after plating 10–30-fold-concentrated water sample (32 out of 67 SRP isolates in Table 3). Some SRP colonies were recovered after direct plating of the non-concentrated water sample (9 out of 67 isolates). The enrichment step allowed the isolation of 26 additional SRP isolates. For six samples, SRPs were obtained only after the enrichment step (Table S3). This was the case in September for four samples from Lake Boufflers and in July for two samples from Lake Riquet. In these six cases, enrichment seems to be the most sensitive method to detect SRP from environmental samples. However, for three samples the SRP species obtained by spreading water directly on CVP plates were not recovered after enrichment (Table S3). This was observed for *D. oryzae* isolated in a June sample and for the *Dickeya* or *Pectobacterium* species found in two August samples. In these three cases, enrichment did not allow SRP recovery from environmental samples. In addition, different species were often recovered from the same sample by direct spreading and after enrichment (Table S3). In the September sample from Lake Page, only *D. oryzae* was obtained by spreading concentrated water sample, and only *D. lacustris* was obtained after enrichment (Table 3). In the September sample from Lake Praillebard, *D. lacustris* was obtained by spreading the concentrated water sample, but *D. parazeae* was obtained after enrichment. In a September sample from Lake Boufflers, only *P. aquaticum* was obtained by spreading concentrated water sample, but both *D. lacustris* and *D. oryzae* were obtained after enrichment. Similarly, in an August sample, *P. quasiaquaticum* was obtained by spreading concentrated water sample while *D. lacustris* was obtained after enrichment. Thus, comparison between SRP isolates obtained with or without enrichment showed that different species could be recovered from the same sample by the two methods (Table 3 and Table S3). Direct water sample spreading and enrichment appeared to be complementary to disclose the diversity of SRP species in lake water.

### 3.6. Pectinolytic Bacteria Detected in the Rhizosphere of Waterside Plants

The *gapA* sequence of 24 rhizosphere isolates was determined, including 17 isolates from bittersweets, 3 from rushes, 2 from reeds and 2 from Bidens. Homologies based on the *gapA* sequence indicated that these isolates consist in 11 *Dickeya* strains (46%), 3 *Pectobacterium* strains (12%) and 10 isolates from other *Enterobacterales* families (42%) (Table 5).

The *gapA* phylogenetic trees suggest that the 14 SRP isolates from rhizosphere samples are distributed in the species *D. chrysanthemi* (7), *D. oryzae* (2), *D. aquatica* (1), *D. lacustris* (1), *P. quasiaquaticum* (1), *P. brasiliense* (1), and *P. versatile* (1) (Figure 1, Figure 2, and Table 5). The ten non-SRP isolates from plant rhizosphere belong to the *Enterobacterales* genera *Klebsiella*, *Raoultella*, *Serratia*, *Kosakonia*, and *Enterobacter* (Table 5, Table S2, and Figure S2).

Focusing on each type of harvested plant, SRPs were identified in the rhizosphere from 7 out of 46 rhizosphere samples (about 15%), including 4 bittersweet samples (out of 22), 2 rush samples (out of 10), 1 Bidens sample (out of 4) and none from reed samples (out of 10) (Table 4). Among the collected plants, the rhizosphere of bittersweets harbored the largest number of SRPs, with 17 isolates, and the highest SRP diversity, with the five species *D. chrysanthemi*, *D. lacustris*, *D. aquatica*, *P. versatile*, and *P. brasiliense*. When individual plants were considered, we observed that the bittersweet rhizosphere sample DA2 contained two SRP genera, *D. chrysanthemi* and *P. brasiliense* (Table 4). Other bittersweet rhizospheres harbored two *Dickeya* species, such as *D. chrysanthemi* and *D. lacustris* in the samples DA3, or *D. chrysanthemi* and *D. aquatica* in the sample DA7. During the winter season, a single SRP member, *D. oryzae* M17, was isolated from rush rhizosphere (Table 5). Unlike the other SRPs, no *D. oryzae* was isolated from bittersweet rhizosphere, while two isolates were obtained from that of rushes, in March and August (Table 5).

With the rhizosphere samples, SRP colonies were mostly recovered after the enrichment step (9/14). The other five isolates were recovered by direct spreading of filtered water in which the roots have soaked (Table 5). No concentration was performed for the rhizosphere samples, due to the high number of colonies growing on CVP. Nine SRP isolates were recovered in September, but most isolates were non-SRP in other surveys, particularly in October (Table 5). For three rhizosphere samples, SRPs were obtained only after the enrichment step (Table 5 and Table S3). In contrast, SRP species were obtained by direct spreading but not after enrichment for four samples (Table 5 and Table S3). This was the case for the sole *D. oryzae* isolate obtained in March from sample J1. Thus, direct water spreading and enrichment appeared to be complementary to disclose SRP species in the rhizosphere samples.

**Table 5:** The 24 rhizosphere isolates identified by phenotypic analysis and *gapA* sequencing.

Isolate Name	Laboratory Collection	Date	Lake	Plant	Sample ID	Selection Method	Aspect of Pits on CVP	Biovar	Identification by *gapA* Sequence
S33	A6071	September 2017	Riquet	Bittersweet	DA1	enrichment	large, deep	_	*Pectobacterium versatile*
S34	A6072	September 2017	Boufflers	Bittersweet	DA2	enrichment	large, deep	_	*Pectobacterium brasiliense*
S35	A6069	September 2017	Boufflers	Bittersweet	DA2	enrichment	large, deep	Bv 5/6	*Dickeya chrysanthemi*
S36		September 2017	Boufflers	Bittersweet	DA2	enrichment	large, deep	Bv 5/6	*Dickeya chrysanthemi*
S37		September 2017	Riquet	Bittersweet	DA3	enrichment	large, deep	Bv 5/6	*Dickeya chrysanthemi*
S38		September 2017	Riquet	Bittersweet	DA3	enrichment	large, deep	Bv 5/6	*Dickeya chrysanthemi*
S39	A6112	September 2017	Riquet	Bittersweet	DA3	enrichment	deep	Bv 10	*Dickeya lacustris*
S40		September 2017	Riquet	Bittersweet	DA3	enrichment	large, deep	Bv 5/6	*Dickeya chrysanthemi*
S41	A6070	September 2017	Riquet	Bittersweet	DA3	enrichment	large, deep	Bv 5/6	*Dickeya chrysanthemi*
M11		March 2018	Riquet	Reed	R1	enrichment	deep	_	*Serratia* sp.
M13		March 2018	Riquet	Reed	R2	filtered water	large, shallow	_	*Serratia* sp.
M17		March 2018	Riquet	Rush	J1	filtered water	large, deep	Bv 3/8	*Dickeya oryzae*
JDA51		July 2018	Boufflers	Bittersweet	DA5	filtered water	deep	_	*Klebsiella pasteurii*
JDA54		July 2018	Boufflers	Bittersweet	DA5	enrichment	deep	_	*Raoultella lignicola*
JDA71		July 2018	Boufflers	Bittersweet	DA7	filtered water	deep	_	*Klebsiella pasteurii*
JDA73	A6195	July 2018	Boufflers	Bittersweet	DA7	filtered water	deep	Bv 5/6	*Dickeya chrysanthemi*
JDA74	A6196	July 2018	Boufflers	Bittersweet	DA7	filtered water	deep	Bv 10	*Dickeya aquatica*
EDA2		October 2018	Riquet	Bittersweet	DA2	enrichment	shallow	_	*Raoultella* sp.
EDA3		October 2018	Riquet	Bittersweet	DA3	enrichment	shallow	_	*Klebsiella pasteurii*
EDA4		October 2018	Riquet	Bittersweet	DA4	enrichment	shallow	_	*Kosakonia* sp.
EJ2		October 2018	Boufflers	Rush	J2	enrichment	shallow	_	*Enterobacter kobei*
AP100	A6343	August 2019	Praillebard	Rush	P10	filtered water	narrow, deep	Bv 3/8	*Dickeya oryzae*
AP140		August 2019	Praillebard	Bidens	P14	filtered water	narrow, deep	Bv 7/9	*Pectobacterium quasiaquaticum*
AP143		August 2019	Praillebard	Bidens	P14	enrichment	shallow	_	*Kosakonia* sp.

### 3.7. Virulence and Adaptation Abilities of SRP Strains

**Figure 3:**
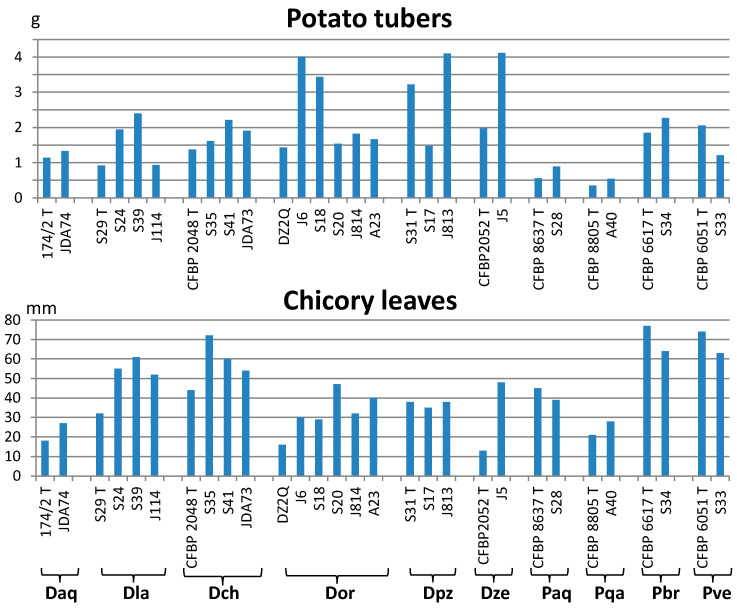
Maceration ability of a subset of SRP isolates.

To analyze the factors involved in their virulence and environmental adaptation, we used a subset of strains belonging to the ten SRP species isolated during this study as well as a set of reference strains (Table S1). To assess their maceration capacity, we infected potato tubers and chicory leaves (Figure 3, Table S4). All isolates were able to macerate plant tissues, although with varying efficiencies. A wide diversity was observed among the strains, both between species and within the same species. No significant differences were observed between strains isolated from water and those from symptomatic plants. On potato tubers, the most effective strains for maceration belonged to the species *D. oryzae*, *D. parazeae* and *D. zeae*. Conversely, the least effective strains were found among the species *P. aquaticum* and *P. quasiaquaticum*. On chicory leaves, the differences between strains were less pronounced than on potato tubers (Figure 3). While the strongest macerations were obtained with members of the species *P. brasiliense*, *P. versatile* and *D. chrysanthemi*; the least effective strains belonged to the species *D. aquatica* and *P. quasiaquaticum*.

**Figure 4:**
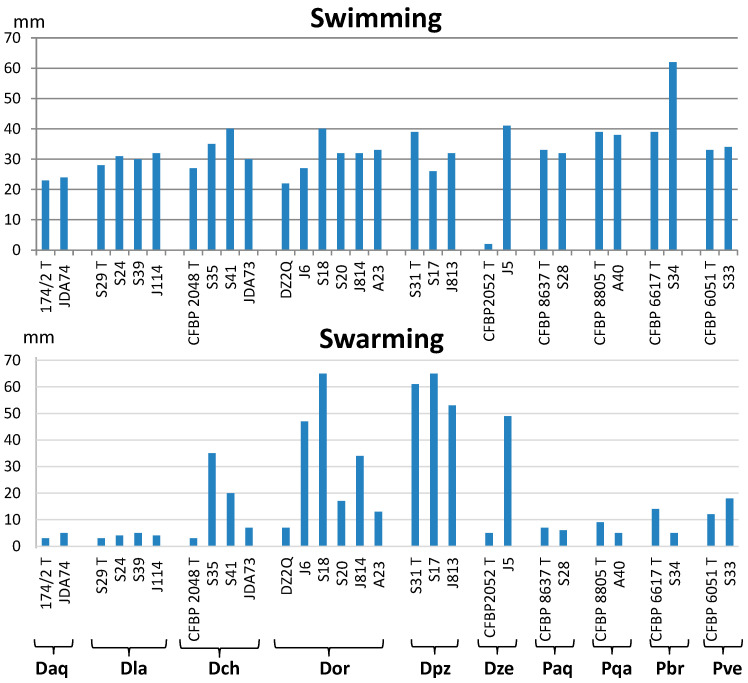
Motility of a subset of SRP isolates.

Since motility is an advantage for phytopathogenic or environmental bacteria, we tested the swimming and swarming mobilities of SRP isolates from water or plant rhizosphere. Reference strains of each species were used for comparison (Figure 4). All strains were able to move by swimming, except the *D. zeae*-type strain which appears to have lost motility, possibly due to prolonged use under laboratory conditions. In contrast, swarming motility varied considerably among the SRP strains. *D. aquatica* and *D. lacustris* strains were incapable of swarming. The *Pectobacterium* strains exhibited no or very low swarming capacity. Only half of the *D. chrysanthemi* strains showed swarming motility. *D. oryzae* strains, except strain DZ2Q, were able to swarm with highly variable efficiencies. *D. parazeae* strains exhibited a high swarming capacity, as did the *D. zeae* strains, with the exception of the type strain (Figure 4).

Temperature is an important factor influencing the growth and survival of environmental bacteria. We tested the growth of SRP isolates at different temperatures, from 20 to 43 °C, (Table 6). Both *Dickeya* and *Pectobacterium* strains showed equivalent growth at moderate temperatures of 20 and 30 °C. At high temperatures, the resistance of *Dickeya* strains was good, as all strains were able to grow at 41 °C and retained reduced growth at 43 °C, with the exception the *D. aquatica* (Table 6). In contrast, *Pectobacterium* strains proved sensitive to high temperatures, none were able to grow at 43 °C, and only *P. brasiliense* strains showed growth at 41 °C (Table 6). Thus, the *Pectobacterium* isolates and the species *D aquatica* appear to be more sensitive to elevated temperature than the other *Dickeya* species isolated from similar habitats (Table 6).

For comparison, the experiment includes reference strains of each species: *D. aquatica* (Daq) 174/2^T^, *D. chrysanthemi* (Dch) CFBP 2048^T^, *D. oryzae* (Dor) DZ2Q, *D. zeae* (Dze) CFBP 2052^T^, *P. aquaticum* (Paq) CFBP 8637^T^, *P. brasiliense* (Pbr) CFBP 6617^T^, *P. quasiaquaticum* (Pqa) CFBP 8805^T^, and *P. versatile* (Pve) CFBP 6051^T^. The reference strains isolated from symptomatic plants are shown in bold letters. The type strains of *D. lactustris* (Dla) S29^T^ and *D. parazeae* (Dpz) S31^T^ were issued from water surveys [10,14]. The capacity of maceration of potato tubers or chicory leaves was measured by the length or weight of rotten tissue, respectively. Data represent the mean values obtained from ten and six replicates for chicory leaves and potato tubers, respectively; these values and standard errors are given in Table S4.

To estimate the swimming and swarming motilities, the diameter of bacterial growth around the inoculation point was measured after growth on specific media. Data represent the mean values obtained from six replicates; these values and standard errors are given in Table S4. The reference strains isolated from symptomatic plants are shown in bold letters.

## 4. Discussion

The affinity of SRPs for aquatic environments has been demonstrated in several previous studies reporting their detection in surface water [33]. However, the numerous changes in SRP classification mean that the taxonomic status of old isolates remains uncertain. For instance, “*Erwinia chrysanthemi*” has been detected in alpine rivers in Australia [34], irrigation water in Spain [35], or irrigation ponds in Florida [36], but this term formerly referred to all the *Dickeya* species. Several *Dickeya* strains isolated from water in Australia, UK, Spain, or Poland were initially classified as *D. zeae* [27,35,37], but many of them were reclassified in the more recently described species *D. oryzae* [13], *D. parazeae* [14] or *D. ananatis* [16] (Table S5).

The frequent association of SRPs with water is confirmed by a significant proportion of SRP strains deposited in international collections and originating from water (Table S5). This raises the question of water as a natural niche for these pectinolytic bacteria. Our objective was to determine whether SRPs can be present in lake water independently of potential contamination from agricultural crops. We therefore carried out surveys in a protected site, comprising four lakes located away from any agricultural practice and fed solely by rainwater. As expected for eutrophic water, the samples showed a highly variable bacterial density, with concentrations in bacteria cultivable on LB medium fluctuating from 2 × 10^2^ to 5 × 10^4^ CFU per mL. Pectinolytic isolates were recovered in 63% of the samples, and the SRP population may reach concentrations higher than 7 × 10^2^ bacteria per liter. Previous reports indicated SRP densities between 10^2^ and 10^3^ bacteria per liter in surface waters in the Netherlands [38]. Higher populations were estimated within lakes and retention ponds in Florida reaching 2.9 × 10^4^ SRP/L [36], 1.4 × 10^5^ SRP/L in Colorado River water [39] and as high as 5.7 × 10^5^ SRP/L in Oregon irrigation water [40]. Thus, large variations of SRP density were observed, depending on the aquatic system and, presumably, on the environmental and experimental conditions. For example, a comparison between SRP isolates obtained with or without enrichment shows that different species were recovered from the same sample using each method (Table 3, Table S3, Table S7). Furthermore, some species were recovered by only one of the methods used (Table S7). While no isolate of *D. aquatica*, *D. zeae*, *D. parazeae* and *P. aquaticum* was found after enrichment, *P. brasiliense* and *P. versatile* were only obtained after enrichment. Enrichment also appears to favor certain *Dickeya* species such as *D. lacustris* and *D. chrysanthemi* (Table S7). Thus, the methodology can influence the estimation of the diversity and relative abundance of SRP species.

In our study of stagnant lake water in La Dombes, both *Dickeya* and *Pectobacterium* species were recovered, corresponding to 82% and 18% of the SRP isolates, respectively. If we do not take into account the isolates obtained after enrichment, the proportion of *Dickeya* and *Pectobacterium* is 79 and 21%, respectively. Eight different SRP species were identified. There was a majority of *D. oryzae* (39%) followed by *D. lacustris* (21%) and *D. parazeae* (19%). The genus *Pectobacterium* mainly included *P. quasiaquaticum* (10%) and *P. aquaticum* (4%). Three other SRP species (*D. chrysanthemi*, *D. zeae*, and *P. brasiliense*) were represented by one or two isolates (1.5–3% each), but even this low representation is indicative of a high diversity of SRPs in lake water. Using similar methods, a large survey of SRPs was performed in water of the river Durance in the South of France [28]. In this previous study, more than 94% of the SRP isolates were assigned to the genus *Pectobacterium* and less than 6% to the genus *Dickeya*. Thus, the relative proportion of each SRP genus seems to greatly differ depending on the water origin, with a majority of *Dickeya* in stagnant lake water and a majority of *Pectobacterium* in running river water. At the species level, different *Pectobacterium* species were observed in the Durance water, including the four recently described species, *P. aquaticum*, *P. quasiaquaticum*, *P. brasiliense* and *P. versatile* [17,18,19]. Notably, members of these species were also identified in our study (Table 3). In the river Durance, the *Dickeya* isolates mostly included *D. oryzae* (69%) and *D. chrysanthemi* (16%), with minor species represented by one isolate of *D. zeae*, *D. parazeae*, *D. solani*, *D. dianthicola* and *D. dadantii* [28] (Table S5). Another study of surface water, limited to the genus *Dickeya*, was conducted in Poland, leading to the isolation of two species, *D. oryzae* and *D. chrysanthemi*, which represent 85% and 15% of the isolates, respectively [27]. In our analysis of lake water, *D. oryzae* was also the most abundant species, representing 47% of the *Dickeya* isolates. Based on these three studies, *D. oryzae* appears to be the predominant *Dickeya* species in surface waters.

We frequently isolated several SRP species from a single sample (15 mL volume) (Table 5). For example, one sample contained up to four species, *D. chrysanthemi*, *D. oryzae*, *P. aquaticum*, and *P. quasiaquaticum.* Another sample contained three species, *D. lacustris*, *D. oryzae* and *P. aquaticum.* Other samples also included isolates from both SRP genera such as *D. lacustris* and *P. brasiliense*, *D. lacustris* and *P. quasiaquaticum*, or *D. oryzae* and *P. quasiaquaticum.* Several samples enclosed two *Dickeya* species, often *D. lacustris* with *D. parazeae*, or *D. oryzae* with *D. parazeae* (Table 5). Thus, this study shows that various SRP species, or even both SRP genera, frequently coexist in small volumes of lake water.

Our study expands to the analysis of plant rhizosphere, indicating that SRPs may be associated to the rhizosphere of riparian plants. Only a few SRP isolates (14) could be isolated from plant rhizospheres due to technical limitations. As plant rhizospheres host a high number of bacteria, including several pectinolytic bacteria from non-SRP species, it was difficult to recover SRP from these samples. Thus, the SRP–rhizosphere association may be underestimated in our study. However, a high SRP diversity was observed in the rhizosphere samples with seven species, namely *D. aquatica*, *D. chrysanthemi D. oryzae*, *D. lacustris*, *D. parazeae*, *P. quasiaquaticum* and *P. versatile*. With 79% *Dickeya* and 21% *Pectobacterium* in the rhizosphere samples, the ratios between the two SRP genera were similar to those observed in water samples (82% *Dickeya* and 18% *Pectobacterium*). However, SRPs from plant rhizospheres include 50% of *D. chrysanthemi*, a significantly higher proportion than in water samples (less than 2%). In contrast, rhizosphere samples contained a reduced proportion of *D. oryzae* or *D. lacustris* (14% and 7%, respectively) in comparison to water samples (39% and 21%, respectively). *D. parazeae* and *P. quasiaquaticum* were not found in plant rhizospheres while they were present at significant levels in water samples (19% and 10%, respectively). Inversely, the two species, *D. aquatica* and *P. versatile*, were observed in plant rhizospheres but not in water. However, these differences may be due to a too small number of isolates from rhizosphere samples. In conclusion, plant roots harbor a high diversity of SRP species. Our preliminary data suggest that *D. chrysanthemi* is more often found in the rhizosphere of riparian plants than in water, but this potential association remains to be confirmed by further observations. Other SRPs such as *D. oryzae*, *D. lacustris*, *D. parazeae*, and *P. quasiaquaticum* were mainly found in water (Figure S3). When individual plant samples were considered, we observed that various *Dickeya* species or even various SRP genera could cohabit in the same plant rhizosphere. For example, some bittersweet rhizosphere samples contained *D. chrysanthemi* and *P. brasiliense*, *D. chrysanthemi* and *D. lacustris*, or *D. chrysanthemi* and *D. aquatica*. Thus, various SRP species and/or the two SRP genera can cohabit in the rhizosphere of the same plant.

At the level of plant species, we collected roots from 47 rhizosphere samples including 22 of *Solanum dulcamara* (bittersweet), 10 of *Juncus effusus* (common rush), 4 of *Bidens tripartita* (three-lobe beggar ticks), and 10 of *Phragmites australis* (common reed). While no SRP bacteria were isolated from the reed samples, they were identified in the rhizosphere of about 20% of the samples from bittersweet, rush and Bidens. The rhizosphere of *S. dulcamara* clearly harbored the greatest number and diversity of SRPs, including the following five species: *D. chrysanthemi*, *D. lacustris*, *D. aquatica*, *P. versatile*, and *P. brasiliense*. Previous studies have reported associations between SRPs and *S. dulcamara*. *Dickeya* strains have been isolated from asymptomatic *S. dulcamara* plants growing in a watercourse in Sweden [33]. *P. carotovorum* strains have been isolated from symptomless roots of *S. dulcamara* plants in Poland [41]. SRPs have also been isolated from *S. dulcamara* plants collected near agricultural fields in Australia [34], the Netherlands [42], and Finland [43]. Based on these reports, it appears that *S. dulcamara* could be a common host for several SRPs, associated with either the roots or the aerial parts, without causing visible symptoms. *S. dulcamara*, a perennial weed of the *Solanaceae* family, is frequently found in wetlands where it may play a role in maintaining SRPs in this type of environment.

The means used by SRPs to withstand the winter period are of great interest. Several previous reports showed a drop in SRP population during the winter season [21]. It was previously suggested that SRPs could overwinter in soil in association with various alternative host plants, maybe in protected locations in the plant roots [33]. Of the 81 SRPs identified in this study, only one isolate was recovered during the winter season. No SRPs were found in water samples collected in March, at temperatures of 3–5 °C. The only SRP strain isolated during this period, belonging to the species *D. oryzae*, originated from a rush rhizosphere. The association of this strain with the rhizosphere may have contributed to its protection against cold, but further ecological information and experimental data are needed to validate the hypothesis that riparian rhizosphere could constitute a winter refuge for some SRPs.

Temperature is undoubtedly a major factor influencing bacterial population density, affecting both growth and survival at extreme temperatures. It has previously been shown that *Pectobacterium* members grow and survive better than *Dickeya* members at low temperature (8 °C) [28]. Our experimental data indicate that, conversely, *Dickeya* members are more resistant to high temperatures (41–43 °C) than *Pectobacterium* members (Table 6). Of the ten SRP species identified in this study, *P. brasiliense* proved more resistant to high temperature than other *Pectobacterium* species, while *D. aquatica* was more sensitive than other *Dickeya* species. Under natural conditions, no SRPs were isolated from water samples at temperatures below 15 °C. High diversity was observed between 15 and 20 °C, with four *Dickeya* species and three *Pectobacterium* species isolated from water or rhizosphere samples (Table S6). Diversity decreased between 20 and 25 °C, with three *Dickeya* species and only one *Pectobacterium* species, *P. quasiaquaticum* (Table S6). At higher temperatures, between 25 and 30 °C, only five *Dickeya* species were isolated, confirming a disadvantage for the genus *Pectobacterium* at high temperatures in the natural environment.

Besides the high temperatures that stagnant lake waters can reach in summer, other lake-specific environmental factors can influence their colonization by SRPs. Even without precise data on the physicochemical parameters of the water, eutrophic lakes are known to have a higher concentration of organic residues than rivers. Nutrient availability promotes SRP growth but also leads to a high concentration of total microbial populations, including many bacteria and fungi with competitive and antagonistic potential. SRPs produce secondary metabolites capable of inhibiting this community [22], but the results of these microbial struggles are difficult to assess. Furthermore, the ability of certain *Pectobacterium* to form biofilms on surfaces [44] can promote their persistence despite river flow. It is therefore clear that various ecological characteristics distinguishing the stagnant waters of lakes from the flowing waters of rivers, as well as the phenotypic properties of each species of SRP, are factors affecting the abundance and diversity of SRPs.

During the survey of pectinolytic bacteria either in water or in plant rhizosphere, we isolated several non-SRP strains. Some isolates belonging to the *Enterobacterales* family were tentatively identified on the basis of the *gapA* sequence. The non-SRP water isolates belong predominantly to the genera *Kosakonia*, *Klebsiella*, and *Serratia*, with a few *Enterobacter* and *Leliotta* members (Table S2, Figure S2). The same genera, with the exception of *Leliotta*, but also including *Raoultella*, were observed among the non-SRP isolates from rhizosphere. Four isolates, found both in water and rhizosphere, most likely belong to a new *Serratia* species, close to *S. fonticola* (Table S2, Figure S2). Two water isolates belong to the species *S. oryzae* whose initial characterization was based on a single strain isolated from rice stems [45].

Four SRP species identified in this study, *D*. *aquatica*, *D. lacustris*, *P. aquaticum* and *P. quasiaquaticum*, have been exclusively found in water up to now and were never described as responsible for crop diseases in natural conditions. However, some virulence capacities were observed for these aquatic SRP species in laboratory conditions [10,44,46]. In contrast, other SRP species recovered both in lake water and plant rhizosphere are known phytopathogens previously isolated from diseased plants, including *D. chrysanthemi*, *D. oryzae*, *D. parazeae*, *D. zeae*, *P. brasiliense* and *P. versatile.* Although significant variability was observed between strains of the same species, this study confirms that strains isolated from water have virulence potential, as they produce plant cell wall-degrading enzymes and are able to induce maceration of plant tissues. These data reinforce the interest of studies providing information on the SRP diversity outside of agricultural systems.

## 5. Conclusions

Surface waters constitute potential reservoirs and dissemination routes for a wide diversity of SRP species. The presence of SRP in water brought up questions about their potential ability to live and survive in aquatic environments. A common hypothesis suggests that SRPs can contaminate water from adjacent agricultural areas, either through rainwater runoff or transport by wetland animals. Another hypothesis suggests that water and aquatic plants constitute a natural niche for SRPs. According to this, some, but not all, SRP species are primarily aquatic microorganisms. This is likely the case for species found exclusively in an aquatic habitat and which may be adapted to this environment, such as *D. aquatica*, *D. lacustris*, *P. aquaticum* and *P. quasiaquaticum.* Other species, such as *D. oryzae*, have been found in high proportions in water, which therefore appears to constitute a favorable environmental niche. Among cultivated plants, the main hosts of *D. oryzae* are rice and corn [14]. Rice cultivation begins with field flooding, a step favorable to contamination by aquatic bacteria. Corn and rice belong to the *Poacea* family whose members are common in natural habitats, particularly wetlands. Unlike other SRPs associated with the rhizosphere of bittersweets, we found *D. oryzae* members only in the rhizosphere of rushes, and it was the only SRP species associated with this plant, maybe suggesting a different host preference. Given that this potential difference in weed preference is based on a limited number of isolates and a small sample size, it remains a preliminary observation that will require further investigation.

Such as *D. oryzae*, several SRP species may be partially aquatic, including *D. chrysanthemi*, *D. parazeae*, and *D. zeae*, frequently observed in surface water. As its name suggests, *P. versatile* is widespread in both plants and aquatic environments. Conversely, SRP species rarely observed in surface water can arrive following contamination from adjacent agricultural areas and persist for some time in this environment. This is the case of phytopathogenic species, such as *D. solani*, *D. dianthicola*, *D. dadantii* or *P. brasiliense* (Table S5). Thus, different SRPs species may exhibit various potentials for life and survival in surface water. In all cases, the aquatic environment would be favorable to interactions between SRPs and higher organisms, whether plants or animals.

## Figures and Tables

**Table 2:** Classification of the pectinolytic isolates by phenotypic profiles.

Enzyme Secretion			Growth Profile					*Dickeya* Biovars *	Number of Isolates		Species Identified in This Study
Pel	Cel	Prt	Mtl	Xyl	Mel	Dara	Tre		Water	Rhizosphere	
+	+	+	+	+	+	+	-	3/8	40	2	*D. oryzae*, *D. parazeae* and *D zeae*
+	+	+	-	-	+	-	-	10	14	2	*D. lacustris* and *D. aquatica*
+	+	+	+	+	+	-	-	5/6	1	7	*D. chrysanthemi*
+	+	+	+	+	-	-	-	none	10	1	*P. aquaticum* and *P. quasiaquaticum*
+	+	+	+	+	+	-	+	none	2	2	*P. brasiliense* and *P. versatile*
+	-	+	+	+	+	+	+	none	2	0	*S. oryzae*

* The classification of *Dickeya* members in biovars is based on profiles determined by the ability to growth with diverse carbon sources. +, positive response; -, negative response. Biovars 1 to 9 were described by Samson et al., 2005 [5]. Biovar 10 was described for the two water species *D. aquatica* and *D. lacustris* [10]. Some biovars (3 and 8; 5 and 6) are grouped together because they differ only in their response to the ADH test, which varies inside a species.

**Table 6:** Growth of a subset of SRP isolates at different temperatures.

			Growth Temperature *		
Strain	Species	Origin	30 °C	41 °C	43 °C
**174/2^T^ NCPPB 4580^T^**	*D. aquatica*	water	+	+	-
JDA74	*D. aquatica*	rhizosphere	+	+	-
**S29^T^ CFBP 8647^T^**	*D. lacustris*	water	+	+	w
S24	*D. lacustris*	water	+	+	w
S39	*D. lacustris*	rhizosphere	+	+	w
J114	*D. lacustris*	water	+	+	w
**CFBP 2048^T^**	*D. chrysanthemi*	*Chrysanthemum*	+	w	w
S35	*D. chrysanthemi*	water	+	w	w
S41	*D. chrysanthemi*	water	+	w	w
JDA73	*D. chrysanthemi*	water	+	w	w
DZ2Q CFBP 8738	*D. oryzae*	Rice	+	+	w
J6	*D. oryzae*	water	+	+	w
S18	*D. oryzae*	water	+	+	w
S20	*D. oryzae*	water	+	+	w
J814	*D. oryzae*	water	+	+	w
A23	*D. oryzae*	water	+	+	w
**S31^T^ CFBP 8716^T^**	*D. parazeae*	water	+	+	w
S17	*D. parazeae*	water	+	+	w
J813	*D. parazeae*	water	+	+	w
CFBP 2052^T^	*D. zeae*	Corn	+	+	w
J5	*D. zeae*	water	+	+	w
**A212-S19-A16 CFBP 8637^T^**	*P. aquaticum*	water	+	-	-
S28	*P. aquaticum*	water	+	-	-
**CFBP 6617^T^**	*P. brasiliense*	Potato	+	+	-
S34	*P. brasiliense*	rhizosphere	+	+	-
**A477-S1-J17 CFBP 8805^T^**	*P. quasiaquaticum*	water	+	-	-
A40	*P. quasiaquaticum*	water	+	-	-
**CFBP 6051^T^**	*P. versatile*	Potato	+	-	-
S33	*P. versatile*	rhizosphere	+	-	-

* The reference strains are shown in bold letters. The OD_600_ was taken as a measure of the bacterial growth after 24 h at each temperature. +, OD > 0.5; w, 0.1 > OD > 0.5; -, OD < 0.1.

## Data Availability

The original contributions presented in this study are included in the article/Supplementary Materials. Further inquiries can be directed to the corresponding author.

## Supplementary Materials

The following supporting information can be downloaded at https://www.mdpi.com/article/10.3390/microorganisms14071459/s1, Table S1: strains selected for detailed phenotypic analysis; Table S2: the non-SRP water and rhizosphere isolates; Table S3: obtention of different SRP species depending on the selection method; Table S4: virulence potential of selected isolates; Table S5: list of *Dickeya* strains isolated from aquatic environments in various studies [47,48,49]; Table S6: SRP species obtained at different temperature ranges; Table S7: effect of the method on the recovery of each genus and species; Figure S1: confirmation of isolate identification by genomic analysis; Figure S2: the *gapA* phylogenetic tree of non-SRP isolates; Figure S3: comparison of species distribution for SRP isolates identified either in water or in the plant rhizosphere.

## Abbreviations

The following abbreviations are used in this manuscript:

CFU	Colony Forming Unit
CVP	Cristal Violet Pectate
PEB	Pectate Enrichment Broth
SRP	Soft Rot *Pectobacteriaceae*

## References

[1] Ma B., Hibbing M.E., Kim H.S., Reedy R.M., Yedidia I., Breuer J., Breuer J., Glasner J.D., Perna T., Kelman A. (2007). Host range and molecular phylogenies of the soft rot enterobacterial genera *Pectobacterium* and *Dickeya*. Phytopathology.

[2] Van der Wolf J.M., Acuña I., De Boer S.H., Brurberg M.H., Cahill G., Charkowski A.O., Coutinho T., Davey T., Dees M.W., Degefu Y.Y., Van Gijsegem F., van der Wolf J.M., Toth I.K. (2021). Diseases caused by *Pectobacterium* and *Dickeya* species around the world. Plant Diseases Caused by Pectobacterium and Dickeya Species.

[3] Mansfield J., Genin S., Magori S., Citovky V., Sriariyanum M., Ronald P., Dow M., Verdier V., Beer S.V., Machado M.A. (2012). Top 10 plant pathogenic bacteria in molecular plant pathology. Mol. Plant Pathol..

[4] Hugouvieux-Cotte-Pattat N., Condemine G., Shevchik V.E. (2014). Bacterial pectate lyases, structural and functional diversity. Environ. Microbiol. Rep..

[5] Samson R., Legendre J.B., Christen R., Fischer-Le Saux M., Achouak W., Gardan L. (2005). Transfer of *Pectobacterium chrysanthemi* (Burkholder et al. 1953) Brenner et al. 1973 and *Brenneria paradisiaca* to the genus *Dickeya* gen. nov. as *Dickeya chrysanthemi* comb. nov. and *Dickeya paradisiaca* comb. nov. and delineation of four novel species, *Dickeya dadantii* sp. nov., *Dickeya dianthicola* sp. nov., *Dickeya dieffenbachiae* sp. nov. and *Dickeya zeae* sp. nov. Int. J. Syst. Evol. Microbiol..

[6] Hugouvieux-Cotte-Pattat N., Jacot-des-Combes C., Briolay J., Pritchard L. (2021). Proposal for the creation of a new genus *Musicola* gen. nov., reclassification of *Dickeya paradisiaca* (Samson et al. 2005) as *Musicola paradisiaca* comb. nov. and description of a new species *Musicola keenii* sp. nov. Int. J. Syst. Evol. Microbiol..

[7] Parkinson N., DeVos P., Pirhonen M., Elphinstone J. (2014). *Dickeya aquatica* sp. nov., isolated from waterways. Int. J. Syst. Evol. Microbiol..

[8] van der Wolf J.M., Nijhuis E.H., Kowalewska M.J., Saddler G.S., Parkinson N., Elphinstone J.G., Pritchard L., Toth I.K., Lojkowska E., Potrykus M. (2014). *Dickeya solani* sp. nov., a pectinolytic plant-pathogenic bacterium isolated from potato (*Solanum tuberosum*). Int. J. Syst. Evol. Microbiol..

[9] Tian Y., Zhao Y., Yuan X., Yi J., Fan J., Xu Z., Hu B., De Boer S.H., Li X. (2016). *Dickeya fangzhongdai* sp. nov., a plant pathogenic bacterium isolated from pear trees (*Pyrus pyrifolia*). Int. J. Syst. Evol. Microbiol..

[10] Hugouvieux-Cotte-Pattat N., Jacot-des-Combes C., Briolay J. (2019). *Dickeya lacustris* sp. nov., a pectinolytic bacterium isolated from lakes in the French region of La Dombes. Int. J. Syst. Evol. Microbiol..

[11] Oulghazi S., Pédron J., Cigna J., Lau Y.Y., Moumni M., Van Gijsegem F., Chan K.-G., Faure D. (2019). *Dickeya undicola* sp. nov., a novel species for pectinolytic isolates from surface waters in Europe and Asia. Int. J. Syst. Evol. Microbiol..

[12] Hugouvieux-Cotte-Pattat N., Brochier-Armanet C., Flandrois J.P., Sylvie Reverchon S. (2020). *Dickeya poaceaphila* sp. nov., a plant-pathogenic bacterium isolated from sugar cane (*Saccharum officinarum*). Int. J. Syst. Evol. Microbiol..

[13] Wang X., He S.W., Guo H.B., Han J.G., Thin K.K., Gao J.-S., Wang Y., Zhang X.-X. (2020). *Dickeya oryzae* sp. nov., isolated from the roots of rice. Int. J. Syst. Evol. Microbiol..

[14] Hugouvieux-Cotte-Pattat N., Van Gijsegem F. (2021). Diversity within the *Dickeya zeae* complex, identification of *Dickeya zeae* and *Dickeya oryzae* members, proposal of the novel species *Dickeya parazeae* sp. nov. Int. J. Syst. Evol. Microbiol..

[15] Hugouvieux-Cotte-Pattat N., Pédron J., Van Gijsegem F. (2023). Insight into biodiversity of the recently rearranged genus *Dickeya*. Front. Plant Sci..

[16] Dobhal S., Hugouvieux-Cotte-Pattat N., Arizala D., Sari G.B., Chuang S.C., Alvarez A.M., Arif M. (2025). *Dickeya ananatis* sp. nov., pectinolytic bacterium isolated from pineapple (*Ananas comosus*). Int. J. Syst. Evol. Microbiol..

[17] Pédron J., Bertrand C., Taghouti G., Portier P., Barny M.A. (2019). *Pectobacterium aquaticum* sp. nov., isolated from waterways. Int. J. Syst. Evol. Microbiol..

[18] Portier P., Pédron J., Taghouti G., Fischer-Le Saux M., Caullireau E., Bertrand C., Laurent A., Chawki K., Oulgazi S., Moumni M. (2019). Elevation of *Pectobacterium carotovorum* subsp. *odoriferum* to species level as *Pectobacterium odoriferum* sp. nov., proposal of *Pectobacterium brasiliense* sp. nov. and *Pectobacterium actinidiae* sp. nov., emended description of *Pectobacterium carotovorum* and description of *Pectobacterium versatile* sp. nov., isolated from streams and symptoms on diverse plants. Int. J. Syst. Evol. Microbiol..

[19] Ben Moussa H., Pedron J., Bertrand C., Hecquet A., Barny M.A. (2021). *Pectobacterium quasiaquaticum* sp. nov., isolated from waterways. Int. J. Syst. Evol. Microbiol..

[20] Broders K., Aspin A., Bailey J., Chapman T., Portier P., Weir B.S. (2022). Building More Resilient Culture Collections: A Call for Increased Deposits of Plant-Associated Bacteria. Microorganisms.

[21] Perombelon M.C.M., Kelman A. (1980). Ecology of the soft rot Erwinias. Annu. Rev. Phytopathol..

[22] Toth I.K., Barny M., Czajkowski R., Elphinstone J.G., Li X., Pédron J., Pirhonen M., Van Gijsegem F., Van Gijsegem F., van der Wolf J.M., Toth I.K. (2021). *Pectobacterium* and *Dickeya*: Taxonomy and evolution. Plant Diseases Caused by Dickeya and Pectobacterium Species.

[23] Waleron M., Misztak A., Waleron M., Jonca J., Furmaniak M., Waleron K. (2019). *Pectobacterium polonicum* sp. nov. isolated from vegetable fields. Int. J. Syst. Evol. Microbiol..

[24] Oulghazi S., Cigna J., Lau Y.Y., Moumni M., Chan K.G., Faure D. (2019). Transfer of the waterfall source isolate *Pectobacterium carotovorum* M022 to *Pectobacterium fontis* sp. nov., a deep-branching species within the genus *Pectobacterium*. Int. J. Syst. Evol. Microbiol..

[25] Pédron J., Guyon L., Lecomte A., Blottière L., Chandeysson C., Rochelle-Newall E., Raynaud X., Berge O., Barny M.-A. (2020). Comparison of environmental and culture-derived bacterial communities through 16S metabarcoding: A powerful tool to assess media selectivity and detect rare taxa. Microorganisms.

[26] Helias V., Hamon P., Huchet E., Wolf J.V.D., Andrivon D. (2012). Two new effective semiselective crystal violet pectate media for isolation of *Pectobacterium* and *Dickeya*. Plant Pathol..

[27] Potrykus M., Golanowska M., Sledz W., Zoledowska S., Motyka A., Kolodziejska A., Butrymowicz J., Lojkowska E. (2016). Biodiversity of *Dickeya* spp. isolated from potato plants and water sources in temperate climate. Plant Dis..

[28] Ben Moussa H., Bertrand C., Rochelle-Newall E., Fiorini S., Pédron J., Barny M.A. (2022). The diversity and abundance of soft rot *Pectobacteriaceae* along the Durance River stream in the Southeast of France revealed by multiple seasonal surveys. Phytopathology.

[29] Meneley J.C. (1976). Isolation of soft-rot *Erwinia* spp. from agricultural soils using an enrichment technique. Phytopathology.

[30] Miller J. (1992). A Short Course in Bacterial Genetics: A Laboratory Manual and Handbook for Escherichia coli and Related Bacteria.

[31] Cigna J., Dewaegeneire P., Beury A., Gobert V., Faure D. (2017). A *gapA* PCR-sequencing assay for identifying the *Dickeya* and *Pectobacterium* potato pathogens. Plant Dis..

[32] Dereeper A., Guignon V., Blanc G., Audic S., Buffet S., Chevenet F., Dufayard J.F., Guindon S., Lefort V., Lescot M. (2008). Phylogeny.fr: Robust phylogenetic analysis for the non-specialist. Nucleic Acids Res..

[33] Toth I.K., van der Wolf J.M., Saddler G., Lojkowska E., Hélias V., Pirhonen M., Tsror Lahkim L., Elphinstone J.G. (2011). *Dickeya* species: An emerging problem for potato production in Europe. Plant Pathol..

[34] Cother E.J., Gilbert R.L. (1990). Presence of *Erwinia chrysanthemi* in two major river systems and their alpine sources in Australia. J. Appl. Bacteriol..

[35] Palacio-Bielsa A., Mosquera M.E.R., Álvarez M.A.C., Rodríguez I.M.B., López-Solanilla E., Rodríguez-Palenzuela P. (2010). Phenotypic diversity, host range and molecular phylogeny of *Dickeya* isolates from Spain. Eur. J. Plant Pathol..

[36] Norman D.J., Yuen J.M.F., Resendiz R., Boswell J. (2003). Characterization of *Erwinia* populations from nursery retention ponds and lakes infecting ornamental plants in Florida. Plant Dis..

[37] Pritchard L., Humphris S., Saddler G.S., Elphinstone J.G., Pirhonen M., Toth I.K. (2013). Draft genome sequences of 17 isolates of the plant pathogenic bacterium *Dickeya*. Genome Announc..

[38] Roozen N.J.M. (1990). Besmetting van Erwinia-Vrij Pootgoed Vanuit Divese Bronnen. Een Literatuuroverzicht (Contamination of Erwinia-Free Seed Potatoes from Various Sources. A Literature Review).

[39] Jorge P.E., Harrison M.D. (1986). The association of *Erwinia carotovora* with surface water in northern Colorado. Am. Potato J..

[40] Cappaert M.R., Powelson M.L., Franc G.D., Harrison M.D. (1988). Irrigation water as a source of inoculum of soft rot *Erwinias* for aerial stem rot of potatoes. Phytopathology.

[41] Fikowicz-Krosko J., Wszalek-Rozek K., Smolarska A., Czajkowski R. (2017). First report on isolation of soft rot *Pectobacterium carotovorum* subsp. *carotovorum* from symptomless bittersweet nightshade occuring in rural area in Poland. J. Plant Pathol..

[42] van Vuurde J.W.L., de Vries P.M. (1992). Detectie van pathogene *Erwinia* spp. van aardappel in oppervlaktewater in de periode 1988–1991 (Detection of pathogenic *Erwinia* spp. from potato in surface water in the period 1988–1991). IPO-DLO Report.

[43] Zhang C.W., Zhang J., Zhao J.J., Zhao X., Zhao D.F., Yin H.Q., Zhang X.X. (2017). *Serratia oryzae* sp. nov., isolated from rice stems. Int. J. Syst. Evol. Microbiol..

[44] Laurila J., Ahola V., Lehtinen A., Joutsjoki T., Hannukkala A., Rahkonen A., Pirhonen M. (2008). Characterization of *Dickeya* strains isolated from potato and river water samples in Finland. Eur. J. Plant Pathol..

[45] Ben Moussa H., Pédron J., Hugouvieux-Cotte-Pattat N., Barny M.A. (2023). Two species with a peculiar evolution within the genus *Pectobacterium* suggest adaptation to a new environmental niche. Environ. Microbiol..

[46] Duprey A., Taib N., Leonard S., Garin T., Flandrois J.P., Nasser W., Brochier-Armanet C., Reverchon S. (2019). The phytopathogenic nature of *Dickeya aquatica* 174/2 and the dynamic early evolution of *Dickeya* pathogenicity. Environ. Microbiol..

[47] Chan K.G., Kher H.L., Chang C.Y., Yin W.F., Tan K.H. (2015). Analysis of pectate lyase genes in *Dickeya chrysanthemi* strain L11, isolated from a recreational lake in Malaysia: A draft genome sequence perspective. Genome Announc..

[48] Ge T., Jiang H., Tan E.H., Johnson S.B., Larkin R.P., Charkowski A.O., Secor G., Hao J. (2021). Pangenomic analysis of *Dickeya dianthicola* strains related to the outbreak of blackleg and soft rot of potato in USA. Plant Dis..

[49] Alic S., Pedron J., Dreo T., Van Gijsegem F. (2019). Genomic characterisation of the new *Dickeya fangzhongdai* species regrouping plant pathogens and environmental isolates. BMC Genom..

