# Supplementary material for: Occurrence of Dickeya and Pectobacterium in Lake Water and the Rhizosphere of Waterside Plants Collected in the French Region La Dombes
Source: Microorganisms. 2026 Jul 2;14(7):1459. doi: 10.3390/microorganisms14071459 (PMC13413596; doi:10.3390/microorganisms14071459)
Supplement: Supplementary file 1 [file microorganisms-14-01459-s001.zip › microorganisms-4355389-supplementary.pdf]

Supporting information

Occurrence of *Dickeya* and *Pectobacterium* in lake water and the rhizosphere of waterside plants collected in the French region La Dombes

Nicole Hugouvieux-Cotte-Pattat and Véronique Utzinger

Table S1. Strains selected for detailed phenotypic analysis

These strains were used for experiments reported in Figures 3 and 4, in Tables 6 and S4.

Strain	Collection N°	Species	Origin
**SRP isolates from lake water or plant rhizosphere (this study)**			
JDA74	CFBP 8722	*Dickeya aquatica*	Bittersweet rhizosphere, 2018
S35	CFBP 8717	*Dickeya chrysanthemi*	Lake Boufflers water, 2017
S41	CFBP 8718	*Dickeya chrysanthemi*	Bittersweet rhizosphere, 2017
JDA73		*Dickeya chrysanthemi*	Bittersweet rhizosphere, 2018
S29^{T}	CFBP 8647^{T}	*Dickeya lacustris*	Lake Boufflers water, 2017
S24		*Dickeya lacustris*	Lake Boufflers water, 2017
S39	CFBP 8649	*Dickeya lacustris*	Bittersweet rhizosphere, 2017
J114	CFBP 8721	*Dickeya lacustris*	Lake Page water, 2018
S20	CFBP 8715	*Dickeya oryzae*	Lake Boufflers water, 2017
S18		*Dickeya oryzae*	Lake Boufflers water, 2017
J6	CFBP 8726	*Dickeya oryzae*	Lake Riquet water, 2017
J814		*Dickeya oryzae*	Lake Riquet water, 2018
A23	CFBP 8725	*Dickeya oryzae*	Lake Praillebard water, 2019
S31^{T}	CFBP 8716^{T}	*Dickeya parazeae*	Lake Boufflers water, 2017
S17		*Dickeya parazeae*	Lake Praillebard water, 2017
J813		*Dickeya parazeae*	Lake Riquet water, 2018
J5		*Dickeya zeae*	Lake Boufflers water, 2017
S28	CFBP 8724	*Pectobacterium aquaticum*	Lake Boufflers water, 2017
S34	CFBP 8720	*Pectobacterium brasiliense*	Bittersweet rhizosphere, 2017
A40	CFBP 8723	*Pectobacterium quasiaquaticum*	Lake Praillebard water, 2019
S33	CFBP 8719	*Pectobacterium versatile*	Bittersweet rhizosphere, 2017
**SRP reference strains**			
NCPPB 4580^{T}	CFBP 8348^{T}	*Dickeya aquatica*	River water, UK, 2012
CFBP 2048^{T}	NCPPB 402^{T}	*Dickeya chrysanthemi*	*Chrysanthemum*, USA, 1956
3937	CFBP 3855	*Dickeya dadantii*	*Saintpaulia ionantha*, France, 1977
DZ2Q	CFBP 8738	*Dickeya oryzae*	*Oryza sativa*, Italy, 2013
CFBP 2052^{T}	NCPPB 2538^{T}	*Dickeya zeae*	*Zeae mays*, USA, 1970
A212-S19-A16^{T}	CFBP 8637^{T}	*Pectobacterium aquaticum*	River water, France, 2016
CFBP 6617^{T}		*Pectobacterium brasiliense*	*Solanum tuberosum*, Brazil, 1999
A477-S1-J17^{T}	CFBP 8805^{T}	*Pectobacterium quasiaquaticum*	River water, France, 2017
CFBP 6051^{T}	NCPPB 3387^{T}	*Pectobacterium versatile*	*Solanum tuberosum*, Netherlands

Table S2. The non-SRP water and rhizosphere isolates

For each isolate, the best match species was determined by BLAST analysis of the *gapA* sequence. This species was assigned to the isolate when the percentage of identity was greater than 99%. Below this threshold, the isolate was defined only at the genus level. This arbitrary threshold was chosen because the *gapA* sequences of SRP strains belonging to the same species exhibit greater than 99% identity.

Isolate	Date	Lake	Temperature or plant	Selection method	Blast best match	% identity	Assignment
A15	August 2019	Praillebard	20.5°C	enrichment	*Kosakonia sacchari*	99.47	*Kosakonia cowanii*
A25	August 2019	Praillebard	21.2°C	enrichment	*Kosakonia sacchari*	99.87	*Kosakonia cowanii*
A63	August 2019	Praillebard	22.9°C	enrichment	*Kosakonia sacchari*	99.74	*Kosakonia cowanii*
A407	August 2019	Praillebard	22.2°C	enrichment	*Kosakonia sacchari*	99.85	*Kosakonia sacchari*
A408	August 2019	Praillebard	22.2°C	enrichment	*Kosakonia sacchari*	99.70	*Kosakonia sacchari*
J132	July 2018	Page	23.9°C	concentration	*Kosakonia cowanii*	99.74	*Kosakonia cowanii*
J711	July 2018	Boufflers	26.4°C	concentration	*Kosakonia cowanii*	99.20	*Kosakonia cowanii*
J811	July 2018	Riquet	28.3°C	concentration	*Kosakonia cowanii*	99.20	*Kosakonia cowanii*
EDA4	October 2018	Riquet	Bittersweet	enrichment	*Kosakonia cowanii*	97.24	*Kosakonia* sp.
AP143	August 2019	Praillebard	Bidens	enrichment	*Kosakonia cowanii*	98.94	*Kosakonia* sp.
P64	October 2018	Boufflers	14°C	filtered water	*Klebsiella pasteurii*	99.87	*Klebsiella pasteurii*
PE64	October 2018	Boufflers	14°C	enrichment	*Klebsiella pasteurii*	99.87	*Klebsiella pasteurii*
EDA3	October 2018	Riquet	Bittersweet	enrichment	*Klebsiella pasteurii*	99.87	*Klebsiella pasteurii*
J712	July 2018	Boufflers	26.4°C	enrichment	*Klebsiella pasteurii*	99.87	*Klebsiella pasteurii*
JDA51	July 2018	Boufflers	Bittersweet	filtered water	*Klebsiella pasteurii*	99.87	*Klebsiella pasteurii*
JDA71	July 2018	Boufflers	Bittersweet	filtered water	*Klebsiella pasteurii*	99.74	*Klebsiella pasteurii*
A12	August 2019	Praillebard	20.5°C	concentration	*Klebsiella grimontii*	100.	*Klebsiella grimontii*
J10	June 2017	Riquet	29.2°C	enrichment	*Klebsiella quasipneumoniae*	100.	*Klebsiella quasipneumoniae*
J11	June 2017	Riquet	29.2°C	enrichment	*Klebsiella quasipneumoniae*	100.	*Klebsiella quasipneumoniae*
J12	June 2017	Riquet	29.2°C	enrichment	*Klebsiella quasipneumoniae*	100.	*Klebsiella quasipneumoniae*
M6	March 2018	Riquet	4.8°C	concentration	*Serratia fonticola*	95.92	*Serratia* sp.
M10	March 2018	Riquet	3.4°C	concentration	*Serratia fonticola*	95.92	*Serratia* sp.
M11	March 2018	Riquet	Reed	enrichment	*Serratia fonticola*	95.92	*Serratia* sp.
M13	March 2018	Riquet	Reed	filtered water	*Serratia fonticola*	95.92	*Serratia* sp.
S32	Sept. 2017	Boufflers	18.6°C	filtered water	*Serratia oryzae*	100.	*Serratia oryzae*
J9	June 2017	Riquet	29.2°C	concentration	*Serratia oryzae*	99.61	*Serratia oryzae*
P8	October 2018	Riquet	13.7°C	filtered water	*Serratia marcescens*	100.	*Serratia marcescens*
A10	August 2019	Praillebard	20.5°C	concentration	*Enterobacter asburiae*	98.55	*Enterobacter* sp.
EJ2	October 2018	Boufflers	Rush	enrichment	*Enterobacter kobei*	99.55	*Enterobacter kobei*
JDA54	July 2018	Boufflers	Bittersweet	enrichment	*Raoultella lignicola*	99.48	*Raoultella lignicola*
EDA2	October 2018	Riquet	Bittersweet	enrichment	*Raoultella ornithinolytica*	97.24	*Raoultella* sp.
P62C	October 2018	Boufflers	14°C	concentration	*Lelliottia amnigena*	98.93	*Lelliottia* sp.

Table S3. Obtention of different SRP species depending on the selection method

Samples from which two selection methods led to the isolation of different SRP species are given.
ND, not done; - no isolate.

**Sample information**			**Selection method**		
**Date**	**Lake**	**Temperature or plant**	**Filtred water**	**Concentrated water**	**Enrichement**
**Water isolates**					
June 2017	Riquet	28.8°C	_	_	*D. parazeae*
June 2017	Riquet	29.2°C	*D. oryzae*	_	_
July 2018	Riquet	28.2°C	_	_	*D. oryzae, D. parazeae*
July 2018	Riquet	28.3°C	_	*D. oryzae, D. parazeae*	_
August 2019	Praillebard	20.5°C	_	*P. quasiaquaticum*	*D. lacustris*
August 2019	Praillebard	22.2°C	*P. quasiaquaticum*	*P. aquaticum, D. oryzae, D. chrysanthemi,*	_
Sept. 2017	Boufflers	18.9°C	_	*P. aquaticum*	*D. lacustris, D. oryzae*
Sept. 2017	Boufflers	18.2°C	_	_	*D. oryzae*
Sept. 2017	Boufflers	17.7°C	_	_	*D. oryzae*
Sept. 2017	Boufflers	17.4°C	_	_	*D. lacustris*
Sept. 2017	Boufflers	17.5°C	_	_	*D. lacustris, P. brasiliense*
Sept. 2017	Page	18°C	_	*D. oryzae*	*D. lacustris*
Sept. 2017	Praillebard	15.6°C	_	*D. lacustris*	*D. parazeae*
**Rhizosphere isolates**					
March 2018	Riquet	Rush J1	*D. oryzae*	ND	_
July 2018	Boufflers	Bittersweet DA7	*D. aquatica, D. chrysanthemi*	ND	_
August 2019	Praillebard	Rush P10	*D. oryzae*	ND	_
August 2019	Praillebard	Bidens P14	*P. quasiaquaticum*	ND	_
Sept. 2017	Riquet	Bittersweet DA1	_	ND	*P. versatile*
Sept. 2017	Riquet	Bittersweet DA3	_	ND	*D. chrysanthemi, P. brasiliense*
Sept. 2017	Boufflers	Bittersweet DA2	_	ND	*D. chrysanthemi, D. lacustris*

Table S4. Virulence potential of selected isolates

The reference strains are shown in bold letters. The ability of isolates to secrete degradative enzymes was assessed on media containing polygalacturonate for pectinase (Pel), carboxymethylcellulose for cellulase (Cel) and skim milk for protease (Prt). In each case, the diameter of haloes appearing after growth around colonies was measured. To estimate the swimming and swarming motilities, the diameter of bacterial growth around the inoculation point was measured after growth on specific media. The capacity of maceration of potato tubers or chicory leaves was measured by the length or weight of rotten tissue, respectively. Motility and virulence data are presented as means ± standard error. For enzyme secretion, data represent the mean values obtained from three replicates; standard errors were less than 1 mm.

		Enzyme secretion			Mobility		Plant maceration	
		Pel	Cel	Prt	Swimming	Swarming	Chicory leaves	Potato tubers
**Strain**	**Species**	**mm**	**mm**	**mm**	**mm**	**mm**	**mm**	**g**
**174/2^{T} NCPPB 4580^{T}**	***D. aquatica***	12	14	11	23±1	3±1	18±5	1.14 ±0.37
JDA74	*D. aquatica*	13	14	11	24±2	5±1	27±11	1.33 ±0.21
**S29^{T} CFBP 8647^{T}**	***D. lacustris***	12	13	12	28±1	3±1	32±16	0.92 ±0.19
S24	*D. lacustris*	13	14	13	31±1	4±1	55±14	1.94 ±0.21
S39	*D. lacustris*	12	14	12	30±3	5±1	61±16	2.40 ±0.39
J114	*D. lacustris*	12	15	13	32±4	4±1	52±11	0.93 ±0.29
**CFBP 2048^{T}**	***D. chrysanthemi***	11	12	15	27±2	3±1	44±12	1.37 ±0.39
S35	*D. chrysanthemi*	12	12	15	35±3	35±5	72±9	1.61 ±0.48
S41	*D. chrysanthemi*	11	13	11	40±4	20±4	60±11	2.21 ±0.47
JDA73	*D. chrysanthemi*	11	13	12	30±3	7±2	54±9	1.91 ±0.42
**DZ2Q CFBP 8738**	***D. oryzae***	18	15	12	22±3	7±2	16+5	1.43+0.26
J6	*D. oryzae*	18	17	11	27±1	47±6	30±6	4.01 ±0.80
S18	*D. oryzae*	16	13	11	40±3	65±11	29±14	3.43±0.89
S20	*D. oryzae*	17	16	13	32±3	17±1	47±11	1.54 ±0.42
J814	*D. oryzae*	16	14	11	32±4	34±7	32±7	1.82 ±0.61
A23	*D. oryzae*	18	16	13	33±5	13±3	40±11	1.67 ±0.36
**S31^{T} CFBP 8716^{T}**	***D. parazeae***	17	13	10	39±5	61±12	38±12	3.22 ±0.74
S17	*D. parazeae*	16	13	10	26±5	65±15	35±16	1.47 ±0.82
J813	*D. parazeae*	16	16	11	32±4	53±4	38±9	4.10 ±0.89
**CFBP 2052^{T}**	***D. zeae***	17	15	9	2±1	5±1	13±2	1.97 ±0.39
J5	*D. zeae*	18	17	12	41±5	49±14	48±16	4.12 ±0.67
**A212-S19-A16 CFBP 8637^{T}**	***P. aquaticum***	10	16	12	33±5	7±1	45±17	0.56±0.20
S28	*P. aquaticum*	10	14	10	32±2	6±1	39±18	0.89 ±0.52
**A477-S1-J17 CFBP 8805^{T}**	***P. quasiaquaticum***	10	15	12	39±5	9±3	21±15	0.35±0.29
A40	*P. quasiaquaticum*	10	15	12	38±3	5±1	28±15	0.54 ±0.28
**CFBP 6617^{T}**	***P. brasiliense***	17	13	14	39±9	14±4	77±18	1.85±0.38
S34	*P. brasiliense*	16	13	15	62±5	5±1	64±23	2.27 ±0.82
**CFBP 6051^{T}**	***P. versatile***	17	14	11	33±4	12±3	74±28	1.21±0.58
S33	*P. versatile*	16	12	12	34±2	18±4	63±16	1.01 ±0.52
**3937**	***D. dadantii***	18	11	10	31±2	51±7	75±16	2.93 ±0.42

Table S5. List of *Dickeya* strains isolated from aquatic environments in various studies

This table includes *Dickeya* strains isolated from water that are correctly identified to the species level, and some strains described in this study. Their numbering in international collections (CFBP, LMG, NCPPB) is indicated when available.

Species	Strain designations	Origin: country, year, plant	Identification criteria	Reference
D. aquatica	174/2^T (NCPPB4580^T, CFBP8348^T, LMG27354^T)	UK 2012, river water	genome	Parkinson et al, 2014
	DW0440	Finland 2005, river water	genome	Parkinson et al, 2014
	CSL RW240	UK, river water	genome	Parkinson et al, 2014
	181/2	UK 2012, river water	*gyrB, infB, rpoB*	Hugouvieux-Cotte-Pattat et al, 2023
	Dw054, Dw0431, Dw0512	Finland 2005, river water	16S rRNA	Hugouvieux-Cotte-Pattat et al, 2023
	JDA74 (CFBP8722)	France 2018, *Solanum dulcamara* rhizosphere	*gapA*	This study
D. chrysanthemi	L11	Malaysia 2014, recreational lake water	genome	Chan et al, 2015
	A604-S21-A17	France 2017, river water	genome	Ben Moussa et al, 2022
	IFB0284, IFB0320, IFB0336	Poland 2011, water	*recA*	Potrykus et al, 2016
	S35 (CFBP8717, LMG32071), S36, S37, S38, S40, S41 (CFBP8718)	France 2017, *Solanum dulcamara* rhizosphere	*gapA*	This study
	JDA73	France 2018, *Solanum dulcamara* rhizosphere	*gapA*	This study
	A406	France 2019, lake water	*gapA*	This study
D. dadantii	A622-S1-A17	France, 2017, river water	genome	Ben Moussa et al, 2022
D. dianthicola	59W	US, 2016, water	genome	Ge et al, 2021
	A260-S21-A16	France, 2016, water	genome	Ben Moussa et al, 2022
	D19-W1	Japan, 2019, water	*recA, dnaX*	Hugouvieux-Cotte-Pattat et al, 2023
D. fangzhongdai	M005	Malaysia, 2013, waterfall	genome	Alic et al, 2019
	M074	Malaysia, 2013, waterfall	genome	Alic et al, 2019
	ND14b	Malaysia, 2013, waterfall	genome	Alic et al, 2019
	MK7	Scotland, river water	genome	Pritchard et al, 2013
D. lacustris	S29^T (CFBP8647^T, LMG308990^T)	France 2017, lake water	genome	Hugouvieux-Cotte-Pattat et al, 2019
	S39 (CFBP8649)	France 2017, *Solanum dulcamara* rhizosphere	*gapA*	This study
	S11, S12 (CFBP8648), S13, S14, S15, S16, S22, S23, S24	France 2017, lake water	*gapA*	This study
	J114 (CFBP8721)	France 2018, lake water	*gapA*	This study
	A13, A14, A16	France 2019, lake water	*gapA*	This study
D. oryzae	S20 (CFBP8715)	France 2017, lake water	genome	Hugouvieux-Cotte-Pattat & Van Gijsegem, 2021
	FVG03	France 2017, water	genome	Hugouvieux-Cotte-Pattat & Van Gijsegem, 2021
	CSL RW192	England, river water	genome	Pritchard et al, 2013
	A642-S2-A17	France 2017, water	genome	Ben Moussa et al, 2022
	A003-S1-M15	France 2015, water	genome	Ben Moussa et al, 2022
	J6 (CFBP8526), J7, J8, J12, J14, S9, S10, S18, S19, S20, S21, S30, S32	France 2017, lake water	*gapA*	This study

	J112, J113, J115, J1112, J121, J122, J123, J211, J814, J818, J8116	France 2018, lake water	*gapA*	This study
	A23 (CFBP8725), A24, A405	France 2019, lake water	*gapA*	This study
	M17	France 2018, *Juncus effusus* rhizosphere	*gapA*	This study
	AP100	France 2019, *Juncus effusus* rhizosphere	*gapA*	This study
	CFBP3707	Israel 1986, water	*gapA*	Hugouvieux-Cotte-Pattat et al, 2023
	A10-S1-M15, A3-S1-M15, A4-S1-M15, A5-S1-M15, A7-S1-M15, A8-S1-M15, A9-S1-M15	France 2015, water	*gapA*	Ben Moussa et al, 2022
	A223-S2-A16, A225-S1-A16, A257-S22-A16, A259-S1-A16	France 2016, water	*gapA*	Ben Moussa et al, 2022
	A443-S1-J17, A509-S20-J17, A515-S2-A17, A515-S2-A17, A524-S2-A17, A545-S2-A17, A552-S2-A17, A557-S2-A17, A587-S2-A17, A631-S20-A17, A632-S20-A17	France 2017, water	*gapA*	Ben Moussa et al, 2022
	IFB0324, IFB0330, IFB0334	Poland 2011, water	*recA*	Potrykus et al, 2016
D. parazeae	S31^T (CFBP8716^T LMG8719^T)	France 2017, lake water	genome	Hugouvieux-Cotte-Pattat & Van Gijsegem, 2021
	A586-S18-A17	France 2017, river water	genome	Ben Moussa et al, 2022
	J4	France 2017, lake water	*gapA*	This study
	J813 J816, J821, J824, J825, J826, J8115, J8120, J831, J832	France 2018, lake water	*gapA*	This study
D. solani	MK16	Scotland, water	genome	Pritchard et al, 2013
	A623-S20-A17	France, 2017, river water	genome	Ben Moussa et al, 2022
D. undicola	2B12^T (CFBP8650^T LMG30903^T)	Malaysia 2014, freshwater lake	genome	Oulghazi et al, 2019
	FVG1-MFV-O17	France, 2017, surface water	genome	Oulghazi et al, 2019
	FVG10-MFV-A16	France, 2016, surface water	genome	Oulghazi et al, 2019
D. zeae	MK19	Scotland, river water	genome	Pritchard et al, 2013
	A661-S21-A17	France 2017, river water	genome	Ben Moussa et al, 2022
	FVG08	France 2017, water	genome	Hugouvieux-Cotte-Pattat & Van Gijsegem, 2021
	J5	France 2017, lake water	*gapA*	This study
	CFBP7084	Spain 2005, irrigation water	*gapA*	Hugouvieux-Cotte-Pattat et al, 2023

Table S6. SRP species obtained at different temperature ranges

Temperature range	Isolates from water samples					Isolates from rhizosphere samples				
	Total number	***Dickeya* species**		***Pectobacterium* species**		Total number	***Dickeya* species**		***Pectobacterium* species**	
		Nb		Nb			Nb		Nb	
**0-5°C**	0					1	1	1 *D oryzae*	0	
**5-15°C**	0					0				
**15-20°C**	23	19	10 *D lacustris*	4	2 *P aquaticum*	9	7	6 *D chrysanthemi*	2	1 *P versatile*
			7 *D oyzae*		2 *P brasiliense*			1 *D lacustris*		1 *P brasiliense*
			2 *D parazeae*							
**20-25°C**	22	15	10 *D oyzae*	7	7 *P quasiaquaticum*	1	0		1	1 *P quasiaquaticum*
			4 *D lacustris*							
			1 *D chrysanthemi*							
**25-30°C**	23	23	11 *D oyzae*	0		2	2	1 *D aquatica*	0	
			11 *D parazeae*					1 *D chrysanthemi*		
			1 *D zeae*							

Table S7. Effect of the method on the recovery of each genus and species

This table gives the number of isolates obtained by each method used to recover the 81 SRP isolates identified from lake water or rhizosphere of riparian plants. The number of isolates is given for each genus (in bold letters) and for each species.

		Method		
	**Direct plating**	**Concentration**	**Enrichment**	**Total**
Dickeya	**11**	**24**	**31**	**66**
D. aquatica	1	-	-	1
D. lacustris	-	2	13	15
D. chrysanthemi	1	1	6	8
D. oryzae	5	17	6	28
D. parazeae	3	4	6	13
D. zeae	1	-	-	1
Pectobacterium	**3**	**8**	**4**	**15**
P. aquaticum	-	3	-	3
P. quasiaquaticum	3	5	-	8
P. brasiliense	-	-	3	3
P. versatile	-	-	1	1

Figure S1. Confirmation of isolate identification by genomic analysis

The Average Nucleotide Identity (ANI) was calculated using the JSpecies Web Server with default parameters. The digital DNA-DNA hybridization (dDDH) values were calculated using the webserver Genome-to-Genome Distance Calculator (GGDC) version 2.1 with the formula 2. Selected isolates were used with the type strains of closely related species: *D. oryzae*, *D. parazeae*, *D. zeae*, *D. chrysanthemi*, *D. aquatica*, *D. lacustris*, *P. brasiliense* and *P. versatile.*

	D. oryzae S20	*D. oryzae* ZYY5^T	*D. parazeae* S31^T	*D. zeae* NCPPB 2538^T	*D. chrysanthemi* S35	*D. chrysanthemi* NCPPB 516	*D. chrysanthemi* S41	*D. chrysanthemi* NCPPB 402^T	*D. aquatica* JDA74	*D. aquatica* 174-2^T	*D. lacustris* S29^T	*D. lacustris* J114	*P. brasiliense* S34	*P. brasiliense* LMG 21371^T	*P. versatile* S33	*P. versatile* NCPPB 3387^T	
D. oryzae S20	100	73.3	57.6	59.9	30.3	30.2	30.7	30.8	23.2	23.2	23.0	23.0	20.8	20.7	20.8	20.7	**dDDH**
D. oryzae ZYY5^T	96.94	100	56.0	58.4	30.0	30.0	30.2	30.1	23.1	23.2	23.2	23.2	21.1	21.1	20.8	20.6	
D. parazeae S31^T	94.14	94.20	100	67,3	30.5	30.3	31.0	30.9	23.2	23.3	23.1	23.0	20.8	20.8	20.7	20.6	
D. zeae NCPPB 2538^T	94.12	94.64	96.00	100	30.3	30.0	30.5	30.5	22.7	22.7	23.0	23.0	20.8	21.1	20.6	20.9	
D. chrysanthemi S35	85.48	85.41	85.76	85.72	100	79.0	69.0	69.0	23.4	23.4	23.4	23.4	20.7	20.5	20.7	20.5	
D. chrysanthemi NCPPB 516	85.75	85.57	85.74	85.90	97.70	100	70.3	70.1	23.5	23.5	23.6	23.6	20.7	20.5	20.7	20.5	
D. chrysanthemi S41	85.74	85.59	86.04	86.15	96.25	96.47	100	93.9	23.5	23.5	23.4	23.4	21.0	20.7	20.9	20.6	
D. chrysanthemi NCPPB 402^T	85.93	85.55	85.98	86.23	96.25	96.45	99.32	100	23.5	23.4	23.3	23.3	20.8	20.6	20.7	20.5	
D. aquatica JDA74	81.93	81.75	82.05	82.29	81.67	81.78	82.02	81.93	100	95.3	32.8	32.9	20.4	20.3	20.5	20.3	
D. aquatica 174-2^T	81.87	81.79	82.14	82.50	81.53	81.83	81.92	82.00	99.57	100	32.9	32.9	20.7	20.6	20.9	20.6	
D. lacustris S29^T	81.69	81.90	81.74	81.74	81.84	81.93	82.05	82.04	87.70	87.70	100	100	20.8	20.6	20.9	20.6	
D. lacustris J114	81.61	81.86	81.78	81.86	81.94	81.97	82.07	82.07	87.66	87.81	100	100	20.8	20.6	20.9	20.6	
P. brasiliense S34	78.76	79.15	78.86	79.03	78.85	78.81	79.10	79.11	78.68	78.78	79.10	79.14	100	68.3	49.9	50.0	
P. brasiliense LMG 21371^T	79.00	79.22	79.05	79.08	78.90	78.89	79.02	78.86	78.78	78.72	79.10	79.07	96.09	100	48.9	49.2	
P. versatile S33	79.07	79.15	79.00	78.95	78.92	78.59	78.90	78.64	78.68	78.67	78.89	78.81	92.42	92.23	100	79.9	
P. versatile NCPPB 3387^T	79.00	78.83	79.02	78.92	78.70	78.82	78.80	78.61	78.69	78.78	78.85	78.82	92.51	92.30	97.82	100	
	**ANI**																

Figure S2. The *gapA* phylogenetic tree of non-SRP isolates

The tree was generated using a ready-to-use pipeline (Phylogeny.lirmm.fr). The bar indicates the number of changes per nucleotide position.

The drop or the leaf after the isolate designation indicate a water or rhizosphere origin, respectively. For rhizosphere isolates, the corresponding plant is indicated near the leaf: Sd, *Solanum dulcamara;* Pa, *Phragmites australis*; Bt, *Bidens tripartite*. The snowflakes indicate strains isolated in winter.

This tree predicts species identifications that cannot be considered certain since they are based on the sequence of the single gene *gapA* whose reliability has not been evaluated in these *Enterobacterales*.

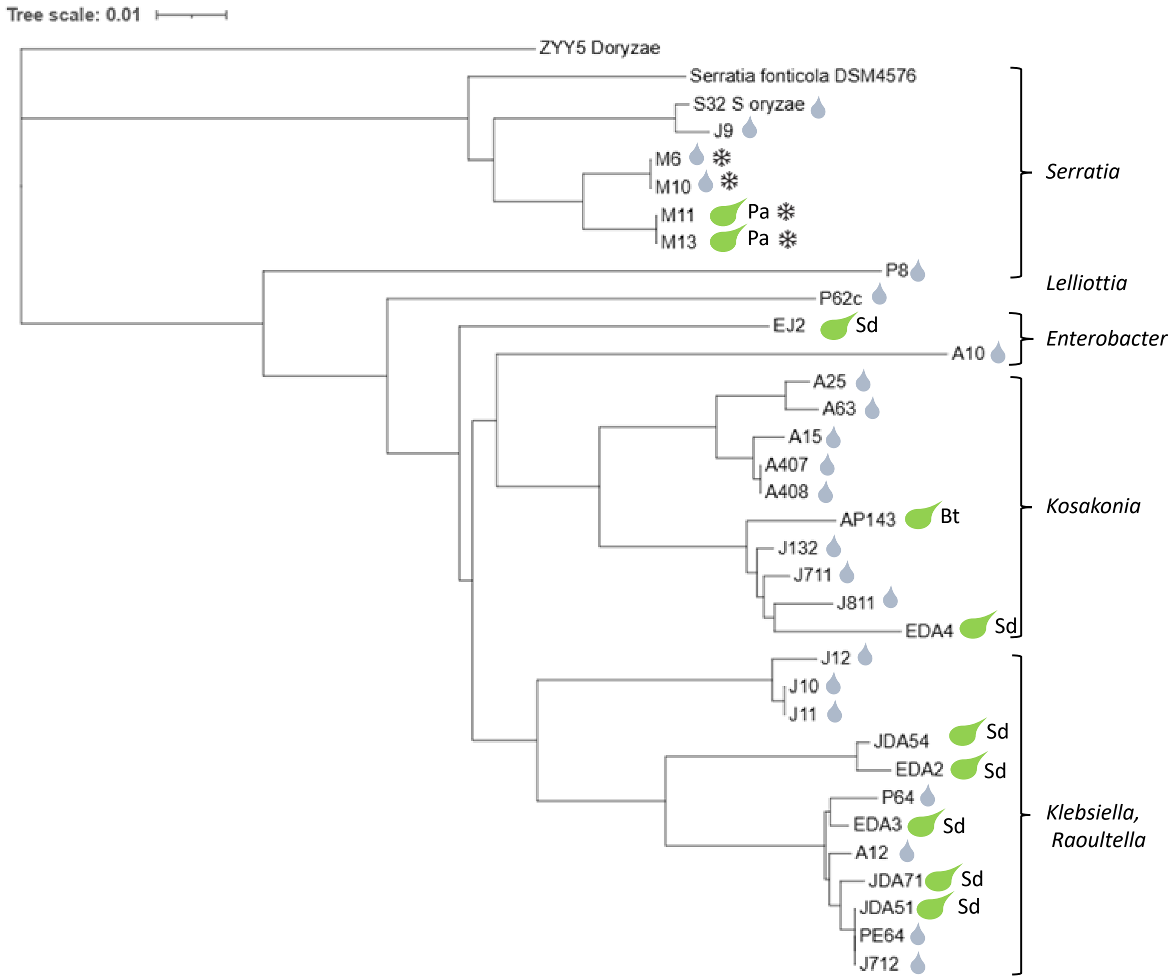

Figure S3. Comparison of species distribution for SRP isolates identified either in water or in the plant rhizosphere

The pie chart representation shows the species distribution of water or rhizosphere isolates in percentage. The table specifies the number of isolates of each species

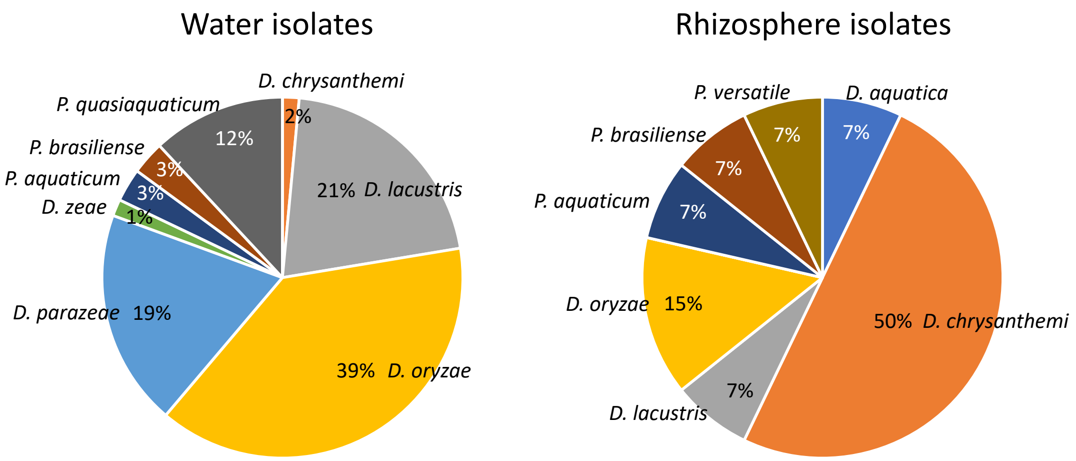

	SRP isolates		
	Total	Water	Rhizosphere
D aquatica	1	0	1
D chrysanthemi	8	1	7
D lacustris	15	14	1
D oryzae	28	26	2
D parazeae	13	13	0
D zeae	1	1	0
P aquaticum	3	2	1
P brasiliense	3	2	1
P quasiaquaticum	8	8	0
P versatile	1	0	1
